# Genome-wide analyses identify KLF4 as an important negative regulator in T-cell acute lymphoblastic leukemia through directly inhibiting T-cell associated genes

**DOI:** 10.1186/s12943-014-0285-x

**Published:** 2015-02-03

**Authors:** Wei Li, Zhiwu Jiang, Tianzhong Li, Xinru Wei, Yi Zheng, Donghai Wu, Lijian Yang, Shaohua Chen, Bing Xu, Mei Zhong, Jue Jiang, Yufeng Hu, Hexiu Su, Minjie Zhang, Xiaojun Huang, Suxia Geng, Jianyu Weng, Xin Du, Pentao Liu, Yangqiu Li, Hudan Liu, Yao Yao, Peng Li

**Affiliations:** Key Laboratory of Regenerative Biology, South China Institute for Stem Cell Biology and Regenerative Medicine, Guangzhou Institutes of Biomedicine and Health, Chinese Academy of Sciences, 190 Kaiyuan Avenue, Science Park, Guangzhou, Guangdong 510530 China; Guangdong Provincial Key Laboratory of Stem Cell and Regenerative Medicine, South China Institute for Stem Cell Biology and Regenerative Medicine, Guangzhou Institutes of Biomedicine and Health, Chinese Academy of Sciences, Guangzhou, 510530 China; Institute of Hematology, Medical College, Jinan University, Guangzhou, 510632 China; Key Laboratory for Regenerative Medicine of Ministry of Education, Jinan University, Guangzhou, 510632 China; Department of Hematology, Nanfang Hospital, Southern Medical University, 510515 Guangzhou, China; Department of Obstetrics and Gynecology, Nan Fang Hospital of Southern Medical University, Guangzhou, 510515 China; School of Pharmacy, Tongji Medical College, Huazhong Unviersity of Science and Technology, 13 Hangkong Road, Wuhan, 430030 China; Shenzhen Institutes of Advanced Technology, Chinese Academy of Sciences, 1068 Xueyuan Avenue, Shenzhen University Town, Shenzhen, 518055 China; Peking University People’s Hospital, Peking University Institute of Hematology, No. 11 Xizhimen South St., Beijing, 100044 China; Department of Hematology, Guangdong Provincial People’s Hospital, Guangzhou, 510500 China; Wellcome Trust Sanger Institute, Hinxton, Cambridge, CB10 1HH England UK; Drug Discovery Pipeline, Guangzhou Institutes of Biomedicine and Health, Chinese Academy of Sciences, 190 Kaiyuan Avenue, Science Park, Guangzhou, Guangdong 510530 China

**Keywords:** KLF4, T-ALL, T cell, NOTCH1, BCL11B, Apoptosis

## Abstract

**Background:**

Kruppel-like factor 4 (KLF4) induces tumorigenesis or suppresses tumor growth in a tissue-dependent manner. However, the roles of KLF4 in hematological malignancies and the mechanisms of action are not fully understood.

**Methods:**

Inducible KLF4-overexpression Jurkat cell line combined with mouse models bearing cell-derived xenografts and primary T-cell acute lymphoblastic leukemia (T-ALL) cells from four patients were used to assess the functional role of KLF4 in T-ALL cells in vitro and in vivo. A genome-wide RNA-seq analysis was conducted to identify genes regulated by KLF4 in T-ALL cells. Chromatin immunoprecipitation (ChIP) PCR was used to determine direct binding sites of KLF4 in T-ALL cells.

**Results:**

Here we reveal that KLF4 induced apoptosis through the BCL2/BCLXL pathway in human T-ALL cell lines and primary T-ALL specimens. In consistence, mice engrafted with KLF4-overexpressing T-ALL cells exhibited prolonged survival. Interestingly, the KLF4-induced apoptosis in T-ALL cells was compromised in xenografts but the invasion capacity of KLF4-expressing T-ALL cells to hosts was dramatically dampened. We found that KLF4 overexpression inhibited T cell-associated genes including NOTCH1, BCL11B, GATA3, and TCF7. Further mechanistic studies revealed that KLF4 directly bound to the promoters of *NOTCH1*, *BCL2*, and *CXCR4* and suppressed their expression. Additionally, KLF4 induced SUMOylation and degradation of BCL11B.

**Conclusions:**

These results suggest that KLF4 as a major transcription factor that suppresses the expression of T-cell associated genes, thus inhibiting T-ALL progression.

**Electronic supplementary material:**

The online version of this article (doi:10.1186/s12943-014-0285-x) contains supplementary material, which is available to authorized users.

## Background

KLF4, also known as GKLF (gut KLF), is a member of the KLF zinc finger-containing transcription factor family [1,2]. Klf4 together with Oct4, Sox2, and c-Myc are widely referred to as ‘Yamanaka factors’ enforced expression of which makes adult cells reprogram into pluripotent stem cells [3]. Consistently, the expression levels of Klf4, Sox2, and Oct4 may need to be decreased during the differentiation of pluripotent cells [4]. Klf4 has critical function in development. Mice homozygous for a null mutation in Klf4 die within a day after birth and show defects in epidermis and colonic epithelial cell differentiation [5]. A recent study reports that the downregulation of Klf4 is required for T cell lineage commitment in mice and Klf4 overexpression blocks T cell development primarily at early stage through suppressing the transcription of several genes that are crucial for early T cell development [6].

T cell development involves progenitor homing and lineage specification and commitment [7]. During early T cell development, several key T cell genes, including *Notch1*, *Bcl11b*, *Gata3*, and *Tcf7* are upregulated [8-11]. T cell development is tightly regulated by key transcription factors, such as Notch1 [12] and Bcl11b [13]. One important mechanism in T cell development is small ubiquitin-like modifier (SUMO) modification because several T cell-associated transcription factors are regulated by SUMO-specific proteases [14]. A previous study identified two SUMO acceptor sites in Bcl11b and demonstrated that prolonged sumoylation resulted in degradation of Bcl11b [15].

T-ALL is thought to result from malignant thymocytes that arise at defined stages of T cell differentiation. Moreover, the expression of certain oncogenes or mutated T cell-specific genes has been closely linked to developmental arrest at particular stages of normal T cell development [16]. Activating mutations of *NOTCH1* were identified in roughly 60% of primary human T-ALLs [17]. Murine T-ALLs studies revealed the presence of acquired gain-of-function *Notch1* mutations at frequencies varying from 30% to 80%, depending on the genetic model [18]. In addition, *BCL11B* mutations are associated with T cell proliferative disorders. The inversion inv(14)(q11.2q32.31) disrupting the *BCL11B* locus has been identified in two cases of T-ALL [19], and monoallelic BCL11B deletions or missense mutations were detected in 9% of T-ALL cases[20]. KLF4 has obtained attention as a negative regulator in T-ALL, because DNA methylation of *KLF4* gene makes its silencing in T-ALL cells and KLF4 overexpression induces apoptosis in ATL-43 T cell line [21]. A recent study identified novel mutations in 3′ untranslated region (UTR) of the KLF4 gene that resulted in loss of miR-2909-mediated regulation in pediatric T-ALL [22]. However, the molecular mechanisms involved in KLF4-induced apoptosis in T-ALL have not been well characterized.

To systematically analyze the genes regulated by KLF4 in T-ALL, we have performed the genome-wide RNA-seq analysis in KLF4 overexpressing Jurkat cells engrafted in immune-compromised NOD-SCID mice. As a negative regulator in human T-ALL in vitro and in vivo, KLF4 was shown to inhibit a variety of T-cell associated genes by directly binding to *NOTCH1* promoter and inducing SUMOylation of BCL11B. Our study thus establishes KLF4 as a critical transcriptional factor directly suppressing T-cell associated transcription factors such as NOTCH1 and BCL11B in malignant T cells.

## Results

### Enforced *KLF4* expression induces apoptosis in Jurkat cells through the BCL2/BCLXL pathway

**Figure 1:**
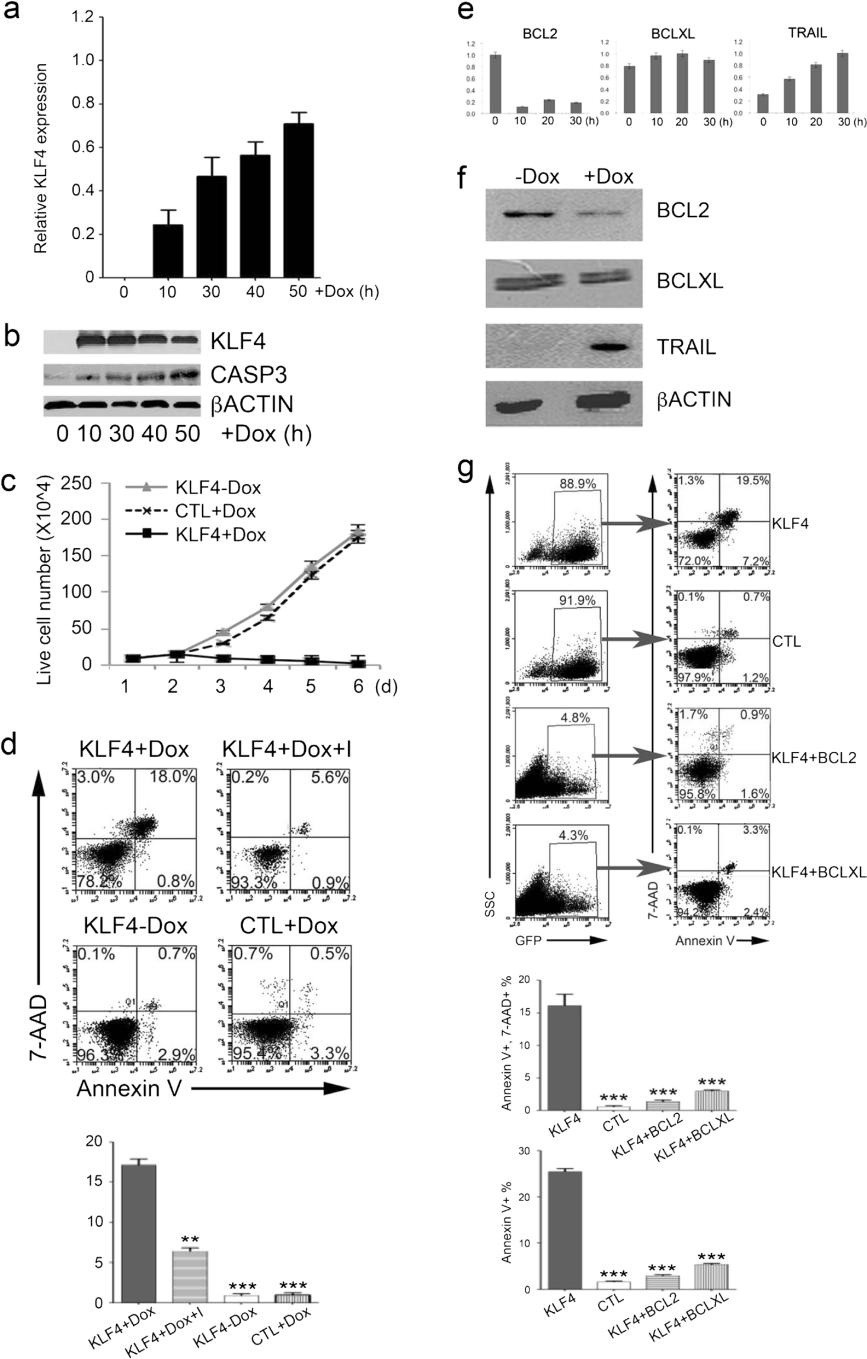
**Enforced expression of** ***KLF4*** **induces apoptosis and suppresses BCL2 in Jurkat cells. (a)** Quantification of KLF4 mRNA levels in TRE-KLF4 Jurkat cells. The results were normalized to the GAPDH mRNA levels and are represented as the mean +/- SEM (n = 3). **(b)** Western blot analysis of KLF4, CASP3, and ACTIN protein levels in TRE-KLF4 Jurkat cells. **(c)** TRE-KLF4 Jurkat cell cultures with (black squares) or without (black diamonds) Dox treatment. TRE-empty Jurkat cell cultures with (grey triangles) Dox treatment. Data are represented as the mean +/- SEM (n = 3). **(d)** TRE-KLF4 Jurkat cells were treated (+Dox) or not (-Dox) with Dox. Z-VAD-FMK (I) and Dox were added at the same time (Dox + I). 48 hours later, cells were subjected to apoptosis assays. Top, representative of flow cytometry profiles of Jurkat cells in apoptosis assays. Bottom, summary of percentages of apoptotic cells (Annexin-V + 7-AAD+) from three independent apoptosis assays. Data are represented as the mean +/- SEM. **P ≤ 0.01 versus bar 1 (for bar 2), *** P ≤ 0.001 versus bar 1 (for bars 3 and 4). **(e)** Quantitative RT-PCR analysis of the expression of selected genes in TRE-KLF4 cells. Data are shown as the mean +/- SEM. **(f)** Western blot analysis of the protein levels of selected apoptosis genes in TRE-KLF4 cells 48 hours post Dox treatment. **(g)** Jurkat cells were transfected with indicated lentiviruses. 48 hours later, GFP+ cells were subjected to apoptosis assays. Top, representative of flow cytometry profiles of Jurkat cells in apoptosis assays. Bottom, summary of percentages of apoptotic cells from three independent assays. Data are represented as the mean +/- SEM. For Annexin-V + 7-AAD+, ***P ≤ 0.001 versus bar 1 (for bars 2-4); For Annexin-V+, ***P ≤ 0.001 versus bar 1 (for bars 2-4).

To investigate the function of KLF4 in Jurkat cells, the TRE-KLF4 and TRE-empty Jurkat cell lines that were constitutively GFP+ were established (Additional files 1 and 2: Figures S1-S2). In TRE-KLF4 cells, the KLF4 overexpression was induced by Doxycycline (Dox) treatment (Figure 1a-b). Dox treatment did not change the expression levels of KLF4 and genes that are related to apoptosis and T cell development in WT Jurkat cells (Additional files 1 and 2: Figure S3). Indeed, we detected massive cell death in Dox-induced TRE-KLF4 cells at 48 hours after Dox treatment, concomitant with the increase of CASP3 (Figure 1b) and accumulation of apoptotic cells, whereas TRE-KLF4 cells without Dox treatment and Dox-treated TRE-empty cells grew well (Figure 1c-d). The protein degradation during cell death might explain why the KLF4 protein level decreased at 50 hours after Dox treatment (Figure 1b). To validate whether KLF4 overexpression induced apoptosis by affecting Caspase activities in Jurkat cells, we treated the Dox-induced TRE-KLF4 cells with Z-VAD-FMK, a pan caspase inhibitor, in an attempt to rescue Jurkat cells from KLF4-mediated apoptosis. Indeed, we found that Z-VAD-FMK treatments reduced the apoptotic rate of Jurkat cells with KLF4 overexpression (Figure 1d). Furthermore, we detected the catalytic activity of CASP3 (Additional files 1 and 2: Figure S4) and the decrease of mitochondrial membrane potential in KLF4 overexpressing Jurkat cells but not in TRE-KLF4 cells without Dox treatment or WT Jurkat cells with Dox treatment (Additional files 1 and 2: Figure S5). These results suggested that the BCL2 pathway was involved in KLF4-induced apoptosis in Jurkat cells.

Upon Dox-induced KLF4 overexpression in Jurkat cells, we measured the expression levels of several genes related to apoptosis at different time points and observed that TRAIL expression was upregulated after Dox treatment, whereas the expression of BCL2 was suppressed, and BCLXL expression remained unchanged (Figure 1e-f). To evaluate whether BCL2 or BCLXL participated in KLF4-induced apoptosis, two lentiviral vectors encoding KLF4-BCL2 and KLF4-BCLXL were constructed and transduced into Jurkat cells (Additional files 1 and 2: Figure S1). Co-expression of BCL2 or BCLXL did not affect KLF4 expression levels in Jurkat cells (Additional files 1 and 2: Figure S6). Jurkat cells transduced with the KLF4 lentivirus demonstrated an apoptotic cell (Annexin-V+, 7-AAD+) frequency of 19.5%, whereas only 0.9% of cells transduced with the KLF4-BCL2 lentivirus underwent apoptosis (Figure 1g). Similarly, the percentage of apoptotic population was reduced to 3.3% when BCLXL was co-expressed with KLF4 (Figure 1g). Thus, enforced expression of BCL2 or BCLXL almost completely rescued Jurkat cells from apoptosis upon KLF4 overexpression, indicating that KLF4 induced apoptosis by suppressing the BCL2 pathway in T-ALL cells.

To exclude the possibility that the effects of KLF4 on Jurkat cells were cell line-specific, we next tested whether KLF4 could induce apoptosis in MOLT4 or CCRF-CEM cells, which are two γ-secretase inhibitors (GSI)-resistant T-ALL cell lines [22] and expressed minimal KLF4 (Additional files 1 and 2: Figure S7). Both cell lines underwent apoptosis upon KLF4 overexpression (Additional files 1 and 2: Figures S8-S9). Furthermore, we confirmed that KLF4 overexpression induced apoptosis in CUTLL1 cells that are sensitive to GSI and did not express KLF4 either [22] (Additional files 1 and 2: Figures S7 and S10). In contrast, KLF4 overexpression did not induce apoptosis either in RL (Additional files 1 and 2: Figure S11), a B cell lymphoma cell line [23], or in K562 (Additional files 1 and 2: Figure S12), a myeloid leukemia cell line [24].

### KLF4 overexpression induces apoptosis in primary T-ALLs

**Figure 2:**
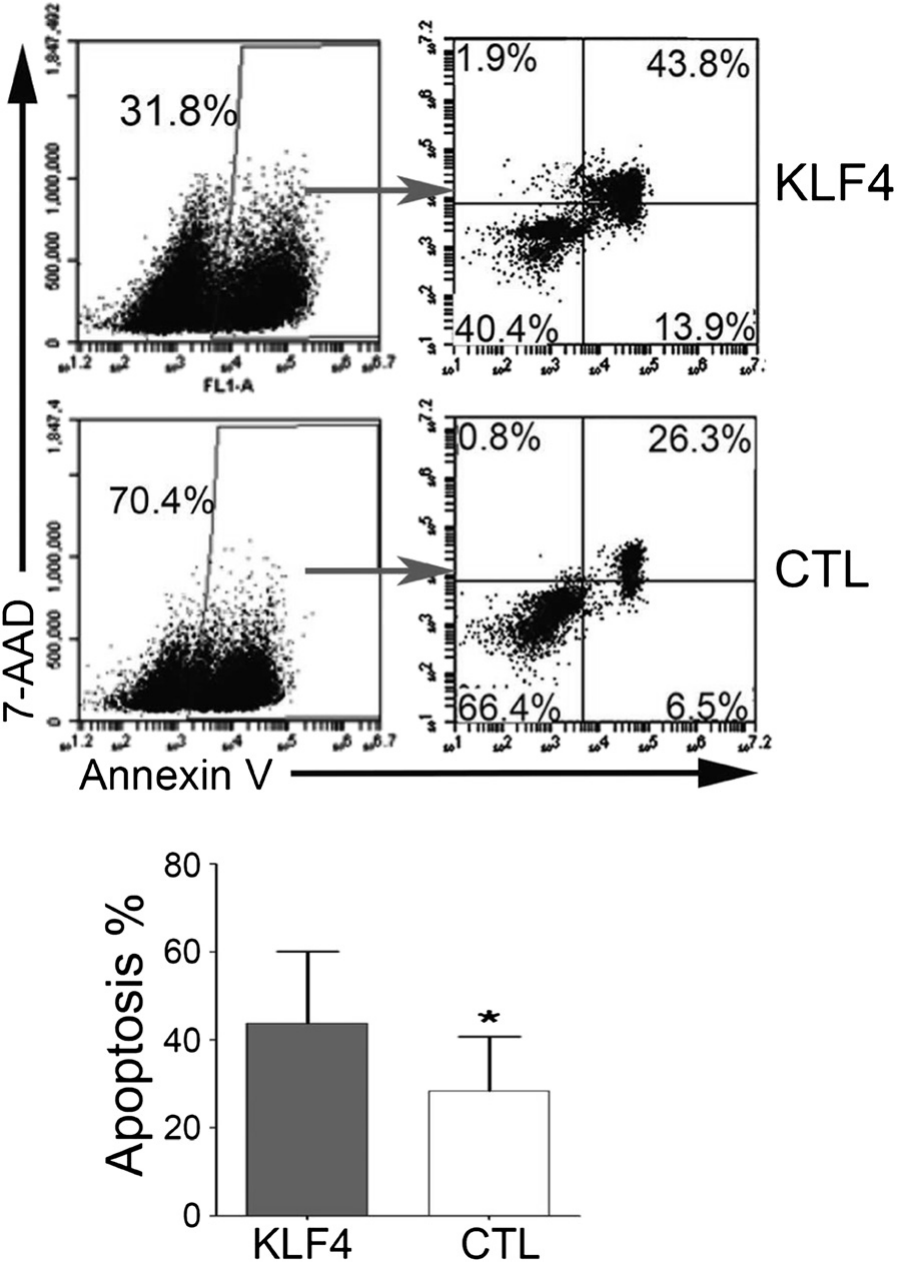
**Effects of KLF4 overexpression on primary T-ALL cells.** Primary T-ALL patient BM samples (n = 3) were transduced with either KLF4-GFP (KLF4) or GFP (CTL) lentiviruses. 72 hours after transduction, GFP+ cells were subjected to apoptosis assays as measured by Annexin-V binding and 7-AAD staining. Top, representative of flow cytometry profiles of primary T-ALL cells in apoptosis assays. Bottom, summary of percentages of apoptotic cells (Annexin-V + 7-AAD+) from three independent apoptosis assays. Data are represented as the mean +/- SEM. *P ≤ 0.05 versus bar 1 (for bar 2).

To validate whether KLF4 induced apoptosis in primary T-ALL cells, we transduced a KLF4-GFP lentivirus into primary T-ALL samples from four patients, in which more than 75% mononuclear BM cells were T-ALL cells (Additional files 1 and 2: Figure S13). KLF4 overexpression caused elevated apoptosis in these cells compared to GFP-transduced controls (Figure 2). To investigate whether there were any mutations in the 3′ UTR of the KLF4 genes that were previously identified in pediatric T-ALL [25], we sequenced the same regions in Jurkat and the two primary T-ALL samples but did not find any mutations (Additional files 1 and 2: Figure S14). These results demonstrate that KLF4 overexpression could induce apoptosis in primary T-ALL cells in vitro.

### KLF4 overexpression reduces aggression of Jurkat cells in vivo

**Figure 3:**
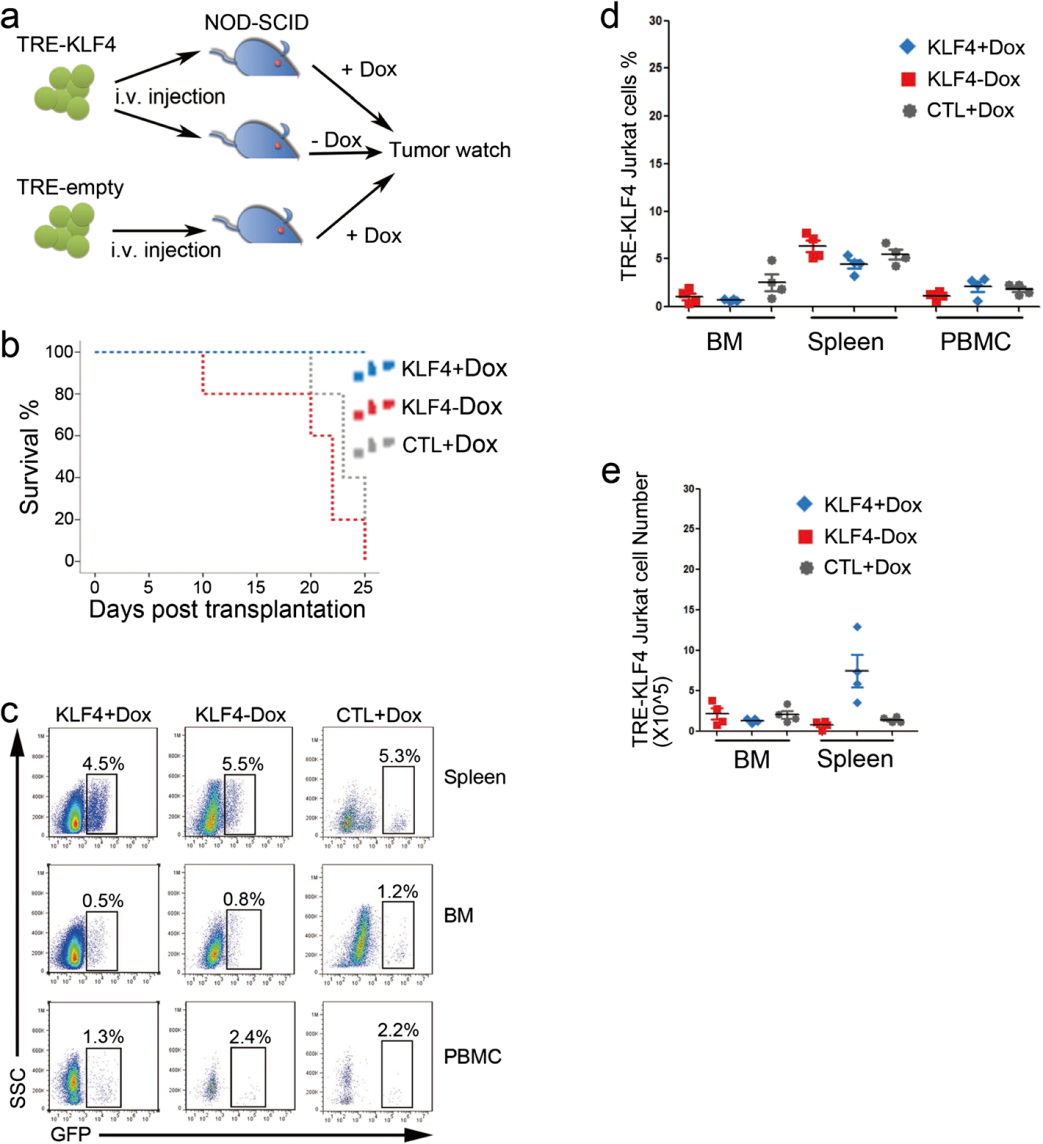
**Overexpression of KLF4 in Jurkat cells in vivo. (a)** Experimental design for studying KLF4 function in Jurkat cells in vivo. TRE-KLF4 Jurkat cells in which KLF4 expression was induced by Dox treatment were intravenously injected into NOD-SCID mice. The injected mice were separated into two groups (n = 15) with Dox treatment or without Dox treatment. In the third group, NOD-SCID mice were injected with TRE-empty Jurkat cells and were subsequently treated with Dox. The three groups of mice were monitored for tumors. Dox was intraperitoneally administered every two days. **(b)** Survival curves for the NOD/SCID mice injected with TRE-KLF4 Jurkat cells. 15 mice were used in each group. Red dots represent Dox-treated mice with injection of TRE-KLF4 Jurkat cells (KLF4 + Dox); Blue dots represent mice injected with TRE-KLF4 cells without Dox treatment (KLF4-Dox); Green dots represent Dox-treated mice with injection of TRE-empty cells (CTL + Dox). **(c)** Two weeks after injection of Jurkat cells, four mice from each group were culled for detection of Jurkat cells. Representative FACS profiles of mononuclear cells of spleen, BM, and peripheral blood from the three groups of mice described in b. **(d)** Summary of percentages of TRE-KLF4 Jurkat cells in spleen, BM, and peripheral blood from the three groups of mice described in b. **(e)** Summary of absolute numbers of TRE-KLF4 Jurkat cells in spleen and BM from the three groups of mice described in b.

To examine the effect of KLF4 overexpression on Jurkat cells in vivo, we injected TRE-KLF4 cells and TRE-empty cells into immunodeficient NOD-SCID mice and started Dox treatments one day after injection of Jurkat cells (Figure 3a). The mice injected with TRE-KLF4 cells without Dox treatment and the mice injected with TRE-empty cells with Dox treatment died within 25 days, while all of the mice that were injected with TRE-KLF4 cells and received Dox treatment started to die two months after injection of Jurkat cells (Figure 3b). To our surprise, the percentages of Jurkat cells in the Dox-treated mice were similar to than that in Dox-untreated mice (Figure 3c-d). In addition, the sizes and cellularity of the spleens in Dox-treated mice were significantly larger than that in Dox-untreated mice (Additional files 1 and 2: Figure S15). Thus, the absolute numbers of Jurkat cells in the spleens of Dox-treated mice were significantly higher than that in Dox-untreated mice (Figure 3e). In addition, cell cycle analysis showed that KLF4 overexpression did not alter the proliferation of Jurkat cells in vivo (Additional files 1 and 2: Figure S16). Taken together, these results indicated that Jurkat cells with KLF4 overexpression survived in vivo but their aggression to hosts was reduced.

**Figure 4:**
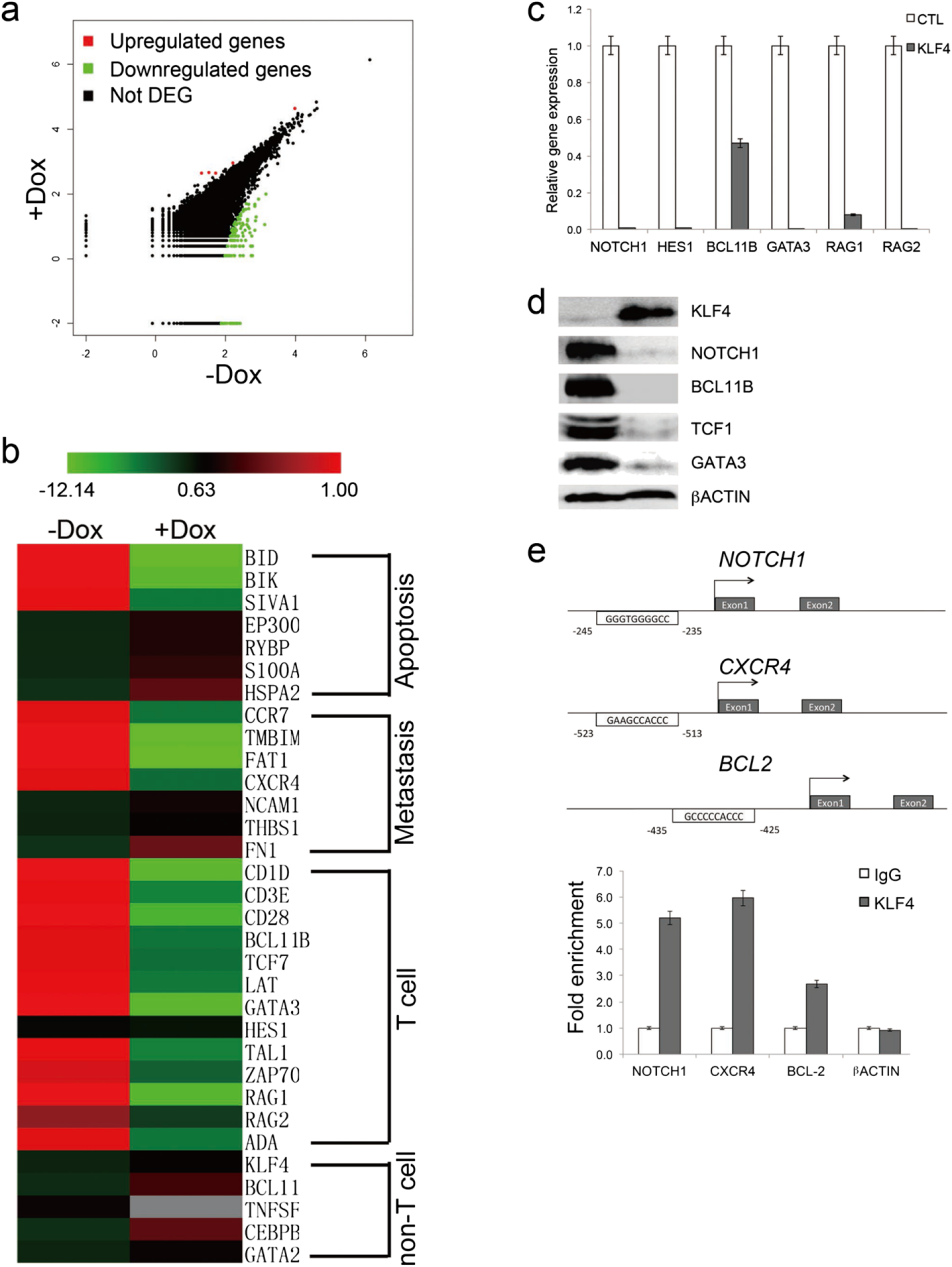
**Downstream targets of KLF4 in T-ALL. (a)** Scatter plots of Dox-treated versus Dox-untreated TRE-KLF4 Jurkat transcriptomes. **(b)** Unsupervised hierarchical cluster analysis of expression levels of 32 genes that are important for apoptosis, metastasis, T cell, or non-T cell lineages in Dox-treated and Dox-untreated TRE-KLF4 Jurkat cells (red, increased expression; green, decreased expression). **(c)** Quantitative RT-PCR analysis of selected gene expression in KLF4 overexpressing Jurkat cells. Data are represented as the mean +/- SEM. **(d)** Western blot detection of T cell specific transcription factors in Jurkat cells 48 hours after KLF4 overexpression. **(e)** KLF4 binds to the promoters of *NOTCH1*, *CXCR4*, and *BCL2* through regions containing conserved KLF4 consensus sequences. Chromatin immunoprecipitates were performed on cross-linked fragmented DNAs prepared from Jurkat cells that forcefully expressed KLF4. Eluted DNAs were then analyzed by qPCR performed with primers flanking putative KLF4-binding sites. The amount of DNA amplified from immunoprecipitated DNAs was normalized to that amplified from input DNA. Data are represented as the mean +/- SEM.

We then compared the gene expression profile of TRE-KLF4 Jurkat cells from Dox-treated mice to that from Dox-untreated mice by RNA-sequencing (RNA-seq) analyses. About 11,000 of the 20860 Refseq genes were detectably expressed (R1 reads per kilo-base exon model per-million reads [RPKM]) in each population after murine transcripts were excluded (Figure 4a). Additional file 3: Table S2 contains a list of genes with greater-than-two fold difference in expression between Dox-treated Jurkat cells and Dox-untreated Jurkat cells. We confirmed the RNA-seq results by quantitative reverse transcription PCR (qRT-PCR) to measure the expression levels of *KLF4*, *BID* [26], *S100A6* [27], *FAT1* [28], *FN1* [29], *CKAP4* [30], genes known to play important roles during apoptosis or cell proliferation (Additional files 1 and 2: Figure S17). With KLF4 overexpression, pro-apoptotic genes including *BID* [26] and *BIK* [31] decreased, while genes involved in anti-apoptosis were upregulated (Figure 4b). Interestingly, *CCR7* [32] that regulates CNS infiltration in T-ALL and *CXCR4* [33,34], which is essential for stem cell and leukemia cell localization were both repressed upon KLF4 overexpression in Jurkat cells (Figure 4b). Furthermore, *TMBIM4* that promotes cell adhesion and migration was downregulated after KLF4 was overexpressed [35]. *FAT1*, a therapeutic target in high-risk preB-ALL, was also suppressed upon KLF4 overexpression [28]. Conversely, cell adhesion proteins including *FN1* [36] and *THBS1* [37] were upregulated after KLF4 was overexpressed (Figure 4b). It was surprising to find that all T cell-associated genes, including T cell specific transcription factors (*BCL11B, TCF7*, and *GATA3*), T cell surface markers (CD1d, *CD3E*, and *CD28*), and TCR-related genes (*ZAP70*, *RAG1*, *RAG2*, and *ADA*) were uniformly silenced in Jurkat cells upon KLF4 overexpression (Figure 4b). Interestingly, *TAL1*, of which aberrant activation is involved in up to 60% of T-ALL cases [38], was severely repressed in Jurkat cells upon KLF4 overexpression (Figure 4b). In contrast, expression of *BCL11A* [39], *CEBPB* [40], and *GATA2* [41] that are important for B, or myeloid cell lineages were upregulated upon KLF4 overexpression (Figure 4b). These results suggested that the tissue homing capacity of Jurkat cells was compromised and T cell transcription program of the T-ALL cell line was disrupted when KLF4 was overexpressed.

### Identification of KLF4 target genes in T-ALL

To validate the results of RNA-seq analysis, we transduced a KLF4-GFP lentivirus and a GFP-only lentivirus as control into Jurkat cells respectively (Additional files 1 and 2: Figures S1 and S18). Forty-eight hours after enforcing the expression of KLF4, we observed that mRNA and protein levels of T cell-associated genes, including NOTCH1, BCL11B, TCF7, and GATA3, decreased significantly upon KLF4 overexpression (Figure 4c-d). To detect potential direct KLF4 target genes in T-ALL, we selected KLF4-repressed candidate genes identified from RNA-seq analysis (Additional file 3: Table S2) that contained KLF4 motif in the promoter regions [42] or were identified by ChIP-seq analysis as direct Klf4 target genes in a mammary epithelial cell line [43] for ChIP validation. Chromatin immunoprecipitates prepared from Jurkat cells, in which the transduction efficiencies of KLF4-GFP lentivirus were about 40% (Additional files 1 and 2: Figure S18), revealed that KLF4 associated with putative binding sites in the promoter regions of *NOTCH1*, *CXCR4*, and *BCL2* (Figure 4e). In contrast, KLF4 did not associated with DNA fragments in the promoters of *βACTIN* (Figure 4e). Taken together, these data suggest that KLF4 downregulated NOTCH1, CXCR4, and BCL2 directly.

### KLF4 induces the SUMOylation and degradation of BCL11B

**Figure 5:**
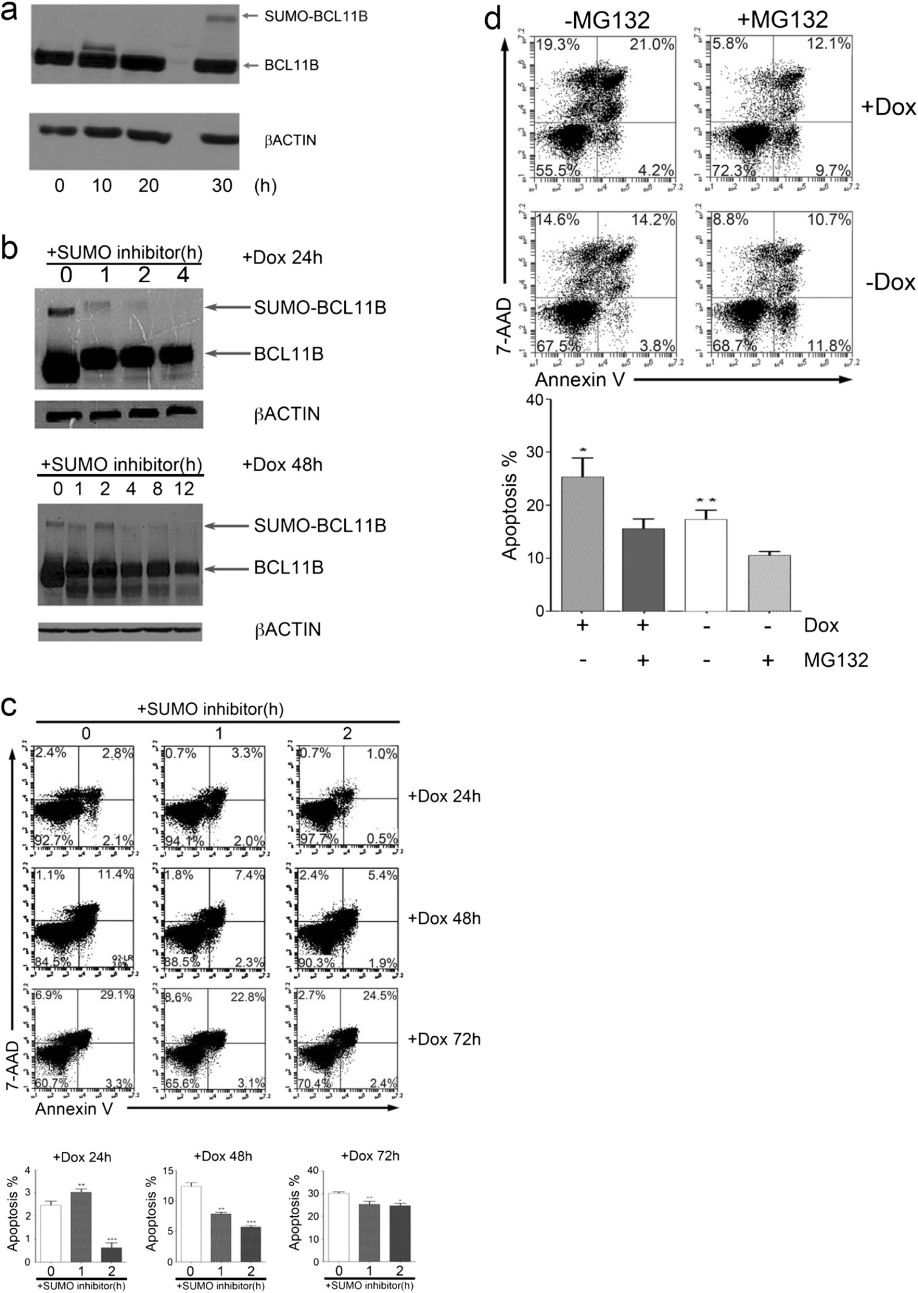
**KLF4 induces the SUMOylation and degradation of BCL11B. (a)** Western blot detection of slowly migrating SUMOylated BCL11B at the indicated time points. **(b)** Western blot analysis of BCL11B SUMOylation status in the presence of SUMO-inhibitor treatment in TRE-KLF4 Jurkat cells. **(c)** Inhibition of SUMOylation reduces KLF4-induced cell death in the Jurkat cell line. Cells were treated with Dox prior to SUMO inhibitor (Calyculin A, 50 nM) addition. At the indicated time points after SUMO inhibitor addition, cells were collected and stained with Annexin-V and 7-AAD for apoptosis detection. Top, representative of flow cytometry profiles of TRE-KLF4 Jurkat cells in apoptosis assays. Bottom, summary of percentages of apoptotic cells (Annexin-V + 7-AAD+) from three independent apoptosis assays. Data are represented as the mean +/- SEM. +Dox 24 h: ** *P* < 0.01 versus bar 1 (for bar 2), *** *P* < 0.001 versus bar 1 (for bar 3); +Dox 48 h: ** *P* < 0.01 versus bar 1 (for bar 2), *** *P* < 0.001 versus bar 1 (for bar 3); +Dox 72 h: ** *P* < 0.01 versus bar 1 (for bar 2), * *P* < 0.05 versus bar 1 (for bar 3). **(d)** Inhibition of proteasome reduces KLF4-induced cell death in the Jurkat cell line. Cells were treated with Dox prior to proteasome inhibitor (MG132, 10 nM) addition. At the indicated time points after MG132 addition, cells were collected and stained with Annexin-V and 7-AAD for apoptosis detection. Top, representative of flow cytometry profiles of TRE-KLF4 Jurkat cells in apoptosis assays. Bottom, summary of percentages of apoptotic cells (Annexin-V + 7-AAD+) from three independent apoptosis assays. Data are represented as the mean +/- SEM. * *P* < 0.05 versus bar 2 (for bar 1), ** *P* < 0.01 versus bar 2 (for bar 3).

Although the transcription levels of *BCL11B* decreased 50% upon KLF4 overexpression (Figure 4c), the levels of BCL11B protein significantly decreased in TRE-KLF4 Jurkat cells 72 hours after the induction of KLF4 overexpression (Figure 4d), while BCL11B protein levels did not change in wild-type Jurkat cells after Dox treatment (Additional files 1 and 2: Figure S19). These results drove us to investigate whether KLF4 affected BCL11B post-translation. SUMOylation is commonly associated with protein degradation; therefore, we speculated that the overexpression of KLF4 might induce the SUMOylation of BCL11B, leading to the degradation of BCL11B protein. We observed SUMOylation of BCL11B at 30 hours after KLF4 was overexpressed in Jurkat cells (Figure 5a). As calyculin A can potently inhibit SUMOylation [15], we found that the SUMOylation of BCL11B was gradually suppressed following calyculin A treatment (Figure 5b). Furthermore, calyculin A partially rescued Jurkat cells from KLF4-induced apoptosis (Figure 5c). Because SUMOylation triggers a secondary signal mediating ubiquitin-dependent degradation by the proteasome [14], we further validated that MG132, a potent proteasome inhibitor [44], could also rescue Jurkat cells from KLF4-induced apoptosis (Figure 5d). Taken together, these results suggested that KLF4 overexpression induced BCL11B protein degradation by SUMOylation.

## Discussion

**Figure 6:**
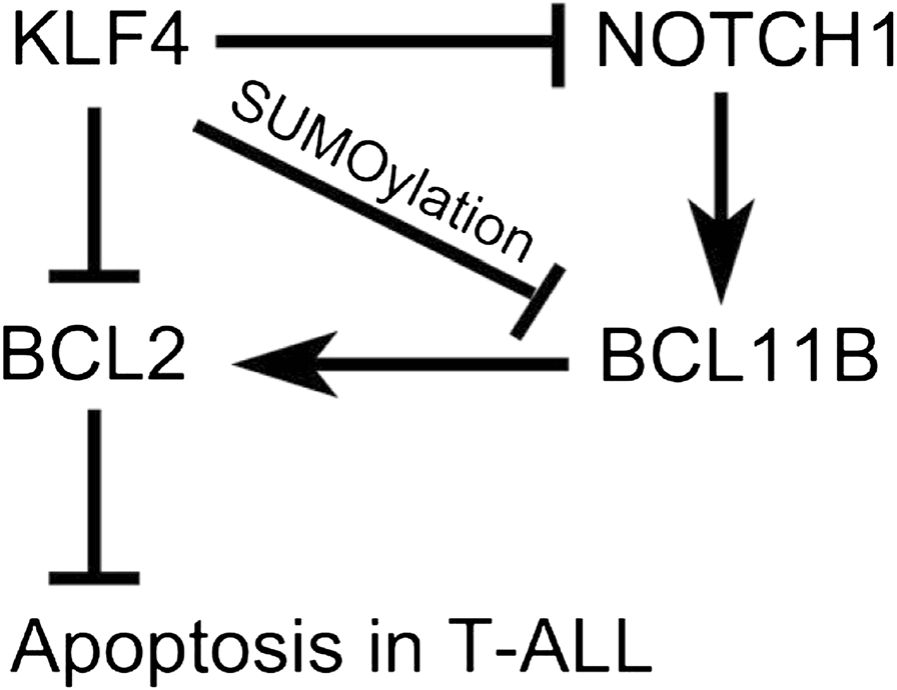
**Working hypothesis of KLF4 function in T-ALL.** KLF4 induces apoptosis in T-ALL cells by directly suppressing BCL2 by directly suppressing *NOTCH1* transcription and inducing BCL11B degradation.

KLF4 acts as a tumor suppressor gene or oncogene depending on cellular contexts. A previous report identified that the *KLF4* locus is hypermethylated in T-ALLs and KLF4 overexpression induced apoptosis in a T-ALL cell line [21]. However the mechanisms of KLF4-induced apoptosis and KLF4 targets in T-ALLs remain unclear. In this study, we used T-ALL as a model system, and demonstrated that the overexpression of KLF4 induced profound apoptosis in four human T-ALL cell lines and primary T-ALL cells in vitro (Figure 6) and increased survival rates in xenografts (Figure 3b). To systematically uncover the transcriptional downstream targets of KLF4, we performed ChIP assays and global gene expression profile analyses and identified that KLF4 directly bound to the promoters of *NOTCH1*, *BCL2*, and *CXCR4* and suppressed their expression in T-ALL. In addition, we demonstrated that KLF4 induced BCL11B degradation by post-translational modification. Consistently, we found that KLF4 negatively regulated human T cell development and homeostasis.

Our studies has clearly demonstrated that KLF4-induced apoptosis was rescued by overexpression of BCL2, which was directly suppressed by KLF4. Interestingly, we noticed that the KLF4 expressing Jurkat cells survived after being injected into immunodeficient mice, suggesting that the KLF4-induced apoptosis was rescued in vivo. Consistently, we observed that pro-apoptotic genes, including *BID*, *BIK*, and *SIVA1*, were downregulated and *EP300*, *RYBP*, *S100A6*, and *HSPA2* that have anti-apoptotic activities were upregulated in KLF4-expressing Jurkat cells two weeks after being injected into hosts. The long term survival of Jurkat cells may explain why secondary effects of KLF4 overexpression, including downregulation of tissue homing genes and upregulation of cell adhesion and non-T cell determination genes, were not observed in cultured Jurkat cells that underwent apoptosis upon KLF4 overexpression within four days. Further investigation is required to identify the molecules or proteins in the in vivo microenvironment that regulated the expression of apoptotic-related genes. This might be helpful to explain why some anti-leukemia drug candidates can efficiently eliminate leukemia cells in vitro but do not work in patients.

The present study also suggested that KLF4 overexpression reduced the invasion capacity of T-ALL cells in hosts. Though there were more Jurkat cells in the Dox-treated mice, these leukemia-bearing mice survived much longer than the Dox-untreated group. It is possible that KLF4-expressing Jurkat cells are less capable of infiltrating into important organs, such as central nervous system, than normal Jurkat cells. T-ALL patients are at an increased risk of CNS relapse [16]. CCR7 is the essential adhesion signal required for the targeting of T-ALL cells in to the CNS [32]. Here, we found that *CCR7* expression was silenced upon KLF4 overexpression in Jurkat cells. In addition, we found *CXCR4*, which promotes T-ALL cells to infiltrate into liver and lung tissues in vivo [34], was directly repressed by KLF4 in Jurkat cells. Furthermore, the expression of *TMBIM4* that increased cell adhesion, spreading, and migration also decreased upon KLF4 overexpression [35]. Downregulation of *FAT1* might also contribute the loss of aggressiveness in T-ALL, because knockdown of *FAT1* in tumor cells results in a drastic inhibition of cell migration and invasion [45]. Thus, KLF4 was identified as a potential repressor of *CCR7*, *CXCR4*, *TMBIM4*, and *FAT1*, the pro-metastasis genes, in T-ALL, which might provide us clues to reduce the invasion capacity of T-ALL in clinics.

Inhibition of NOTCH1 signaling with GSIs has been proposed a molecular targeted therapy for T-ALL. However, GSIs seem to have limited anti-leukemic activity in human T-ALL and are associated with severe gastrointestinal toxicity [46]. Thus, alternative anti-NOTCH1 approaches are in demand for improving T-ALL therapies. We found that KLF4 directly bound to *NOTCH1* promoter, and suppressed NOTCH1 signaling and its downstream targets (Figure 6). Although a recent report showed that Klf4 suppresses Notch signaling in murine angiogenesis [47], for the first time, we identified that NOTCH1 was a direct target of KLF4 for transcription suppression in T-ALLs. Followed by repression of NOTCH signaling, BCL11B, TCF7, and GATA3, the downstream targets of NOTCH signaling, were all decreased upon KLF4 overexpression in T-ALL. In addition, downregulation of T cell surface markers and TCR signaling and upregulation of non-T cell transcription factors in KLF4-expressing Jurkat cells could be caused by the silence of these T cell transcription factors. It will be worthy to develop a chemical approach to initiate endogenous KLF4 expression for inhibition of NOTCH1 signaling in T-ALL.

*BCL11B* mutations are associated with T cell proliferative disorders, even though it is arguable whether BCL11B acts as tumor suppressor or oncogene in T-ALL. The inversion inv(14)(q11.2q32.31) disrupting the *BCL11B* locus was identified in two cases of T-ALL [19], and monoallelic BCL11B deletions or missense mutations were detected in 9% of T-ALL cases [20]. Furthermore, deletions within *BCL11B* were found in irradiation-induced lymphomas in mice, suggesting that BCL11B is a haploinsufficient tumor suppressor. However, BCL11B overexpression was found in the acute type of adult T-cell leukemia/lymphoma and the majority of T-ALL cell lines [19,48]. It was previously reported that the downregulation of BCL11B by RNAi triggered human T-ALL cells to undergo apoptosis through the BCL2/BCLXL pathway, implicating that BCL11B acts as an oncogene [49,50]. Consistently, we found that overexpression of KLF4, an indicated tumor suppressor gene in T-ALL, promoted the SUMOylation and degradation of BCL11B in T-ALL, suggesting that BCL11B acted as an oncogene in T-ALL. However, it remains unclear whether KLF4 directly induces BCL11B SUMOylation or degradation.

## Conclusions

In summary, this study demonstrated that KLF4 directly repress NOTCH1 and serves as a negative regulator in human T-ALL and T cell development. Therefore, reactivation of KLF4 in T-ALL cells may pave a new road for T cell leukemia therapy.

## Methods

### Cell culture

All T-ALL cell lines were obtained from the American Type Culture Collection (ATCC, Manassas, Virginia, USA) and maintained in RPMI-1640 media (Gibco, New York, USA) with 10% fetal bovine serum (FBS, Hyclone, Utah, USA). The 293 T cells used for lentivirus packaging were kindly provided by Professor Duanqing Pei and maintained in DMEM media (Hyclone, Utah, USA) with 10% FBS. OP9-DL1 cells were obtained from Dr. J. C. Zuniga Pflucker (University of Toronto, Toronto, Canada). All cells were incubated at 37°C in 5% CO_2_.

All primary samples were obtained with informed consent for research purposes, and the procedures were approved by the Research Ethics Board of GIBH. T-ALL clinical samples were obtained with informed consent from donors, and related studies were approved by the Institutional Review Boards at Jinan University Medical School. In all four T-ALL patients, more than 80% of PBMC were T-ALL cells.

### Reagents

All chemicals were from Sigma Chemicals (Munich, Germany) unless otherwise specified. The pan caspase inhibitor Z-VAD-FMK was purchased from Beyotime (Jiangsu, China). Antibodies to BCL11B (ab18465), NOTCH1 (ab27526), GATA3 (ab61168), TCF7 (ab30961), KLF4 (ab106629) and SUMO1 (ab32058) were purchased from Abcam (Cambridge, UK). Anti-FLAG, anti-beta-ACTIN, and anti-TRAIL antibodies were purchased from Cell Signal Technologies (Beverly, MA, USA). Antibodies against BCL2, and BCLXL were obtained from Beyotime (Jiangsu, China). All secondary antibodies used in this study were purchased from Sigma (Munich, Germany). A second-generation lentiviral plasmid and related helper plasmids were kindly provided by Professor Xiaoping Chen. The human KLF4 coding sequence was PCR-amplified (Additional file 4: Table S1) and inserted into the *Eco*RI and *Spe*I restriction sites in the pWPXLD lentiviral vector. The correctly ligated plasmid was then sequenced and prepared for virus packaging. Lentiviral vectors encoding the rtTA element and KLF4 driven by a TRE promoter (TRE-KLF4) were generously provided by Professor Duanqing Pei.

### Mice

Animal experiments were performed in the Laboratory Animal Center of Guangzhou Institutes of Biomedicine and Health (GIBH), and all animal procedures were approved by the Animal Welfare Committee of GIBH. NOD-SCID mice were bred and maintained in SPF-grade cages and provided with autoclaved food and water. Mice at 8-12 weeks of age were given 3 Gy of sublethal irradiation and intravenously injected with 5 × 10^7^ Jurkat cells 6 hours following the irradiation. Mice were monitored daily for signs of weight loss or lethargy, and leukocytes from the peripheral blood were subjected to fluorescence-activated cell sorting (FACS) analysis.

### Analysis of gene expression

The sequencing reads were mapped to the mouse RefSeq-RNA reference sequence (downloaded from http://hgdownload.cse.ucsc.edu/downloads) using the FANSe 2 algorithm (http://bioinformatics.jnu.edu.cn/software/fanse2/) with the parameters − L85 − E3 − U0 − S10 [51]. Reads mapped with tophat2 were associated with genes using the custom Perl scripts that allowed no more than 2 unmapped bases. Cufflinks (version 2.1.1) were used to identify reads that were consistent with the annotated genes download from Ensembl database (http://asia.ensembl.org/downloads.html) [52]. These genes were quantified using RPKM method [53]. For small genes (less than 200 bps) a minimum of 10 mappable reads were required. The mappable reads were imported into DEGseq software package to calculate the up-/down-regulation of genes comparing among PL08, 3 T3, and PL08-M with a cut-off value that 2-fold ratio in RPKM and fisher-test FDR of less than 0.05, respectively [54].

### ChIP assay

ChIP was performed as previously described in a previous study [55]. Briefly, 1 × 10^7^ Jurkat cells were transduced by a KLF4-GFP lentivirus, crosslinked with 1% formaldehyde, and subjected for sonication to generate 500-750 bp DNA fragments. The soluble DNA fragments were immunoprecipitated by anti-KLF4 antibody (ab106629, Abcam, United Kingdom) or normal rabbit IgG (ab190495, Abcam, United Kingdom). The immunoprecipitated DNA was eluted and amplified by quantitative PCR on Bio-Rad CFX96 PCR equipment with the following primers specific for the NOTCH1, CXCR4, BCL2 or an irrelevant genomic region (βACTIN) (Additional file 4: Table S1). All ChIP experiments were independently prepared and repeated at least for three times.

### Statistical analysis

Data were analyzed using GraphPad Prism 4 with Student’s *t*-test. P values less than 0.05 were considered statistically significant.

For detailed information, please find it in Additional file 5.

## Additional files

Additional file 1:
**Supplementary Figures.**
Additional file 2:
**Supplementary Figure Legends.**
Additional file 3: Table S2. RNA-sequencing results. Additional file 4: Table S1. Primers used in this study. Additional file 5:
**Supplementary Methods.**

## Abbreviations

T-ALL: T-cell acute lymphoblastic leukemia
ChIP: Chromatin immunoprecipitation
SUMO: Small ubiquitin-like modifier
GSI: γ-secretase inhibitors
qRT-PCR: Quantitative reverse transcription PCR
RNA-seq: RNA-sequencing
