# Supplementary material for: Genome-wide analyses identify KLF4 as an important negative regulator in T-cell acute lymphoblastic leukemia through directly inhibiting T-cell associated genes
Source: Mol Cancer. 2015 Feb 3;14:26. doi: 10.1186/s12943-014-0285-x (PMC4350611; doi:10.1186/s12943-014-0285-x)

Supplementary Figure 1

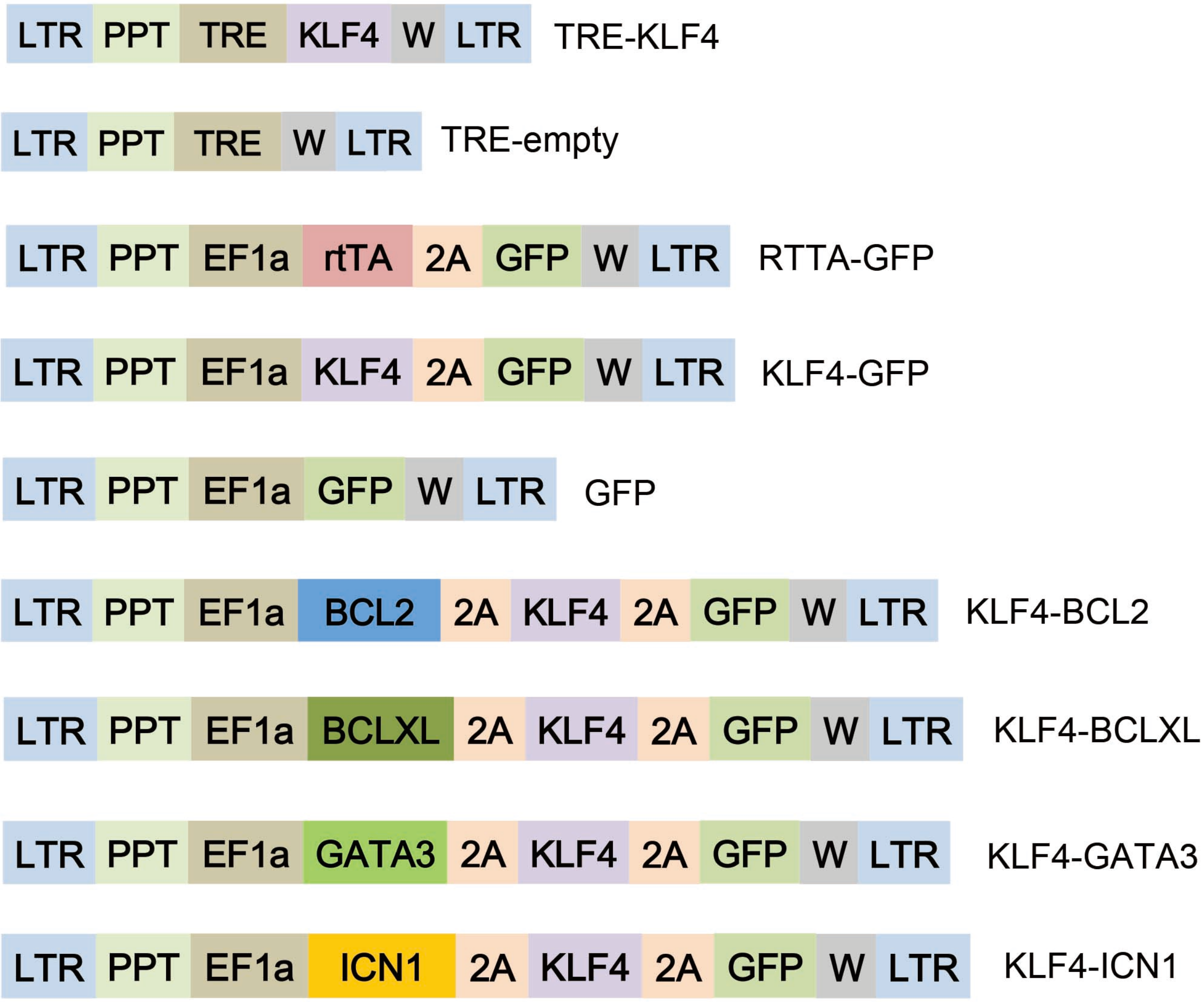

Supplementary Figure 2

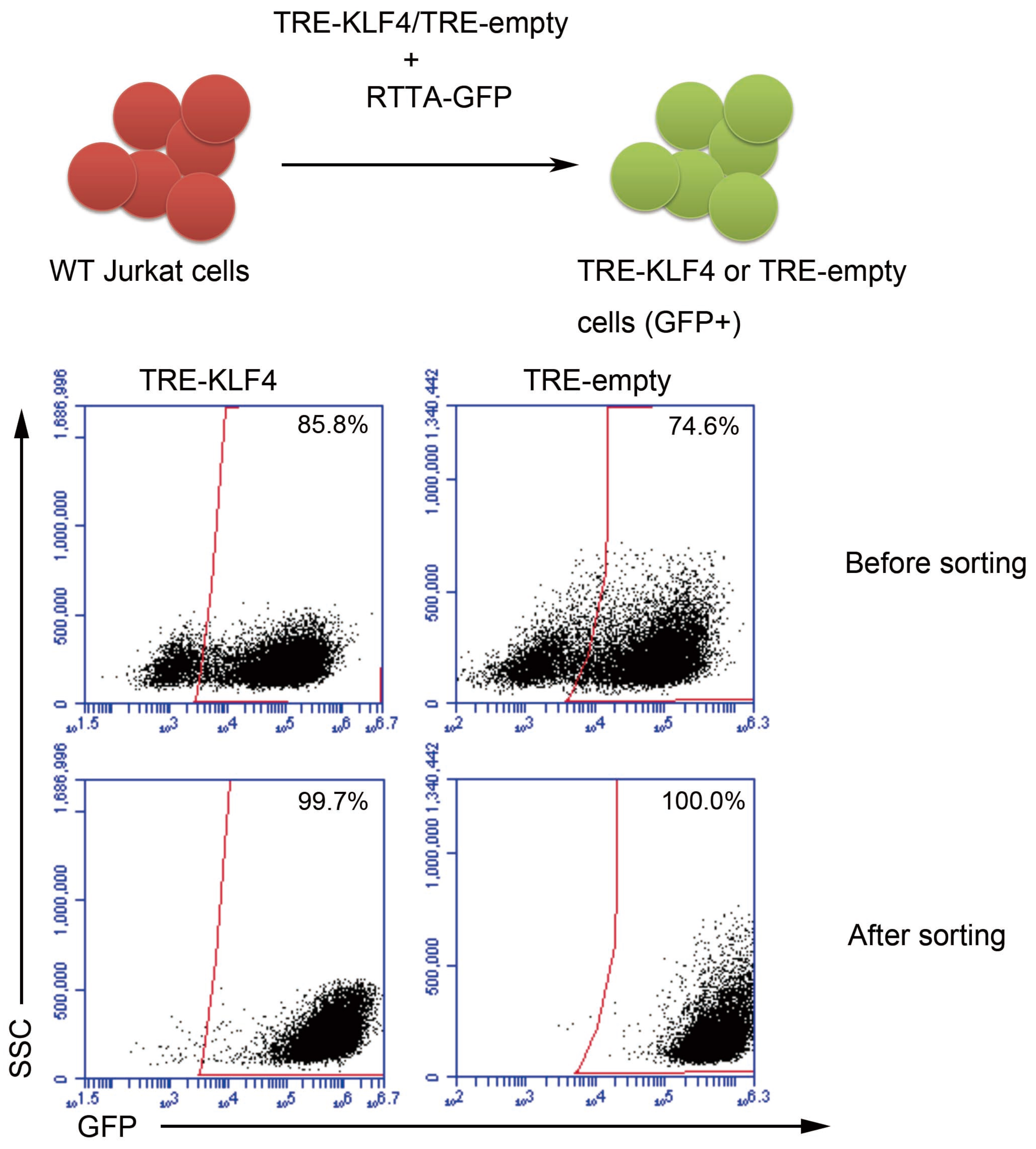

Supplementary Figure 3

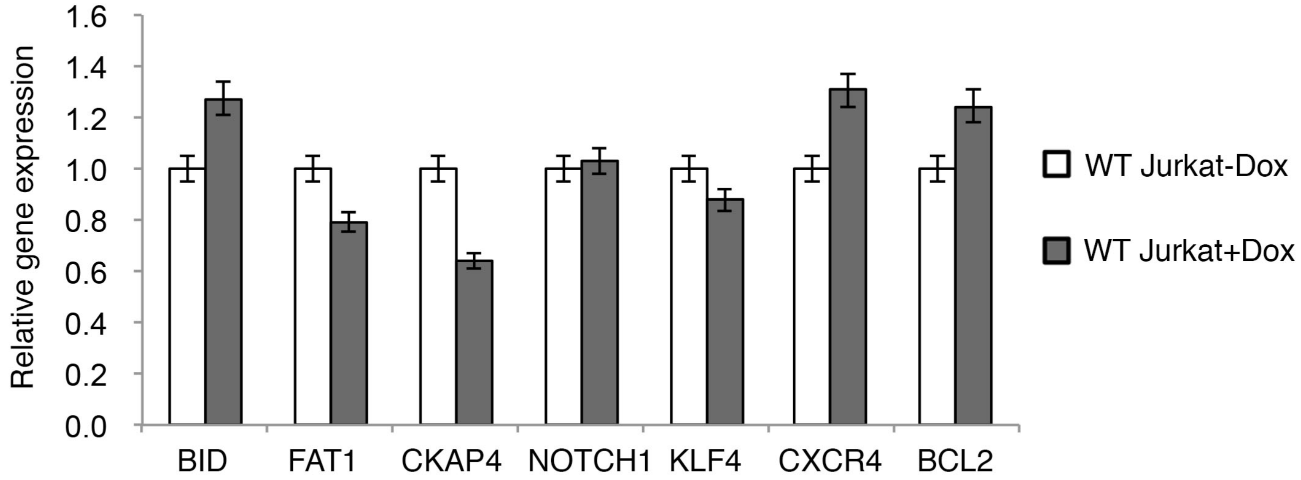

Supplementary Figure 4

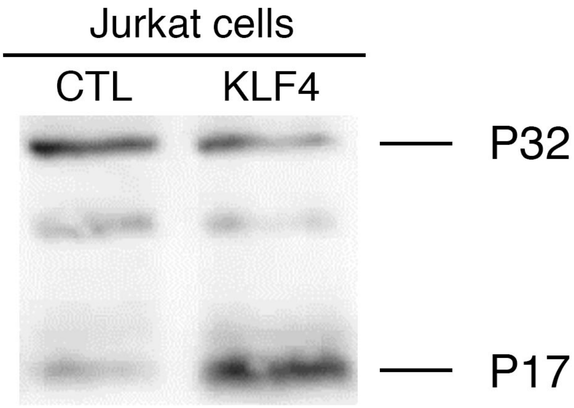

Supplementary Figure 5

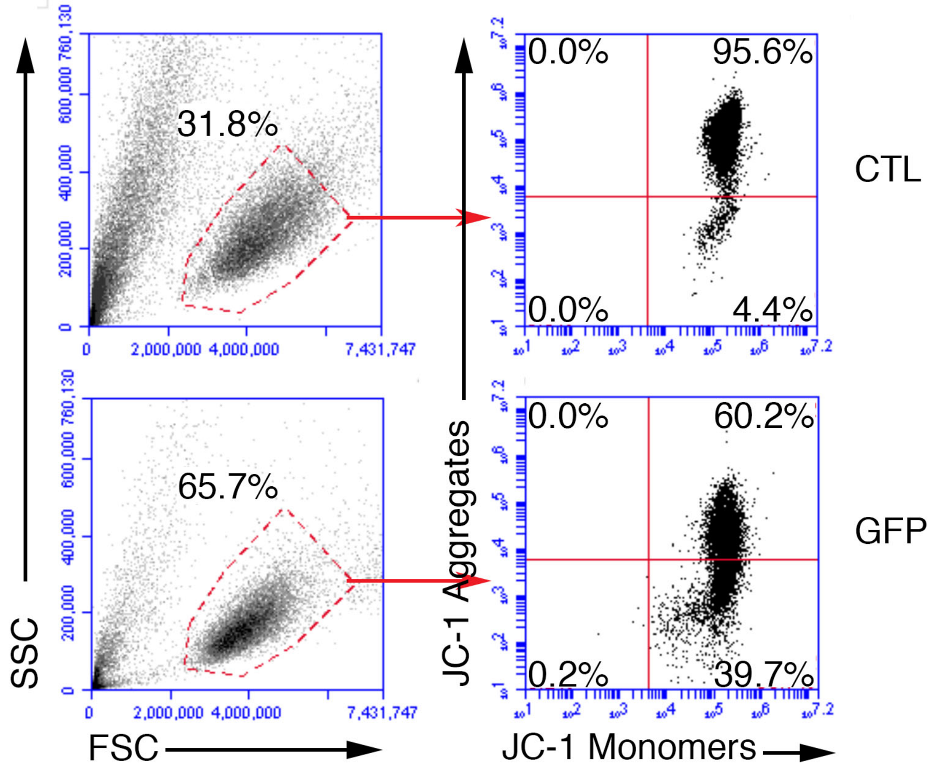

Supplementary Figure 6

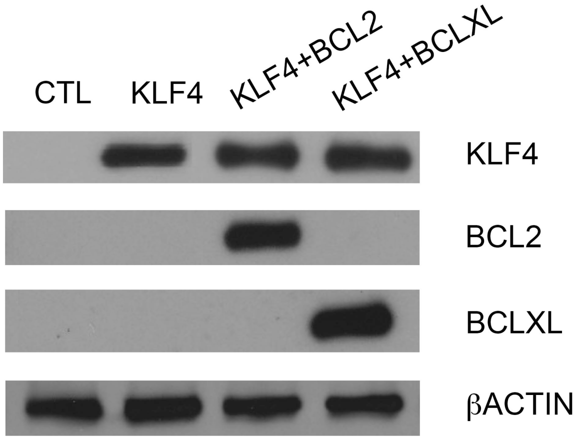

Supplementary Figure 7

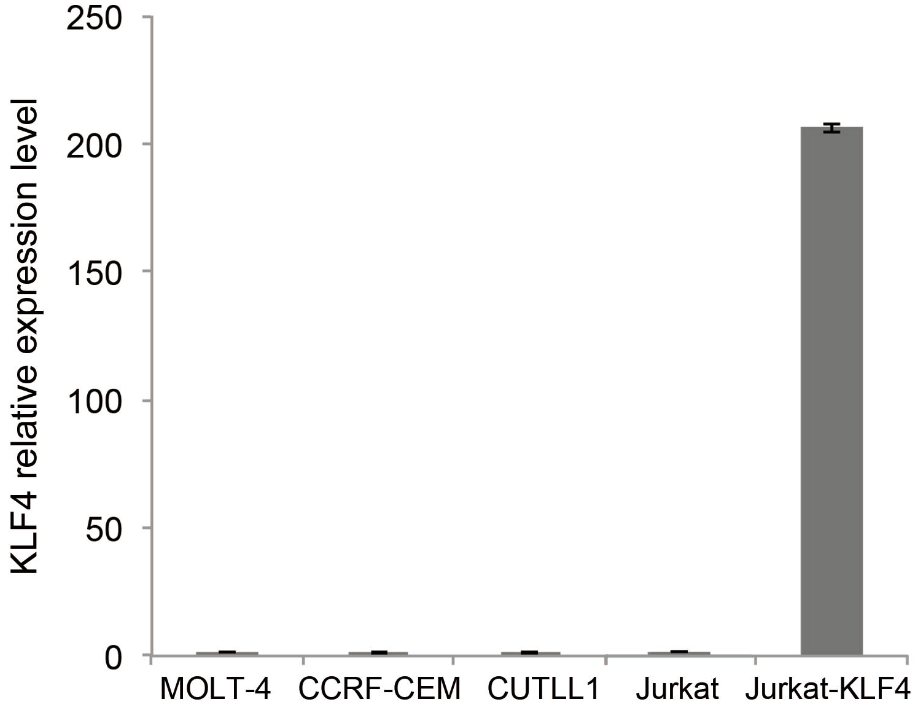

Supplementary Figure 8

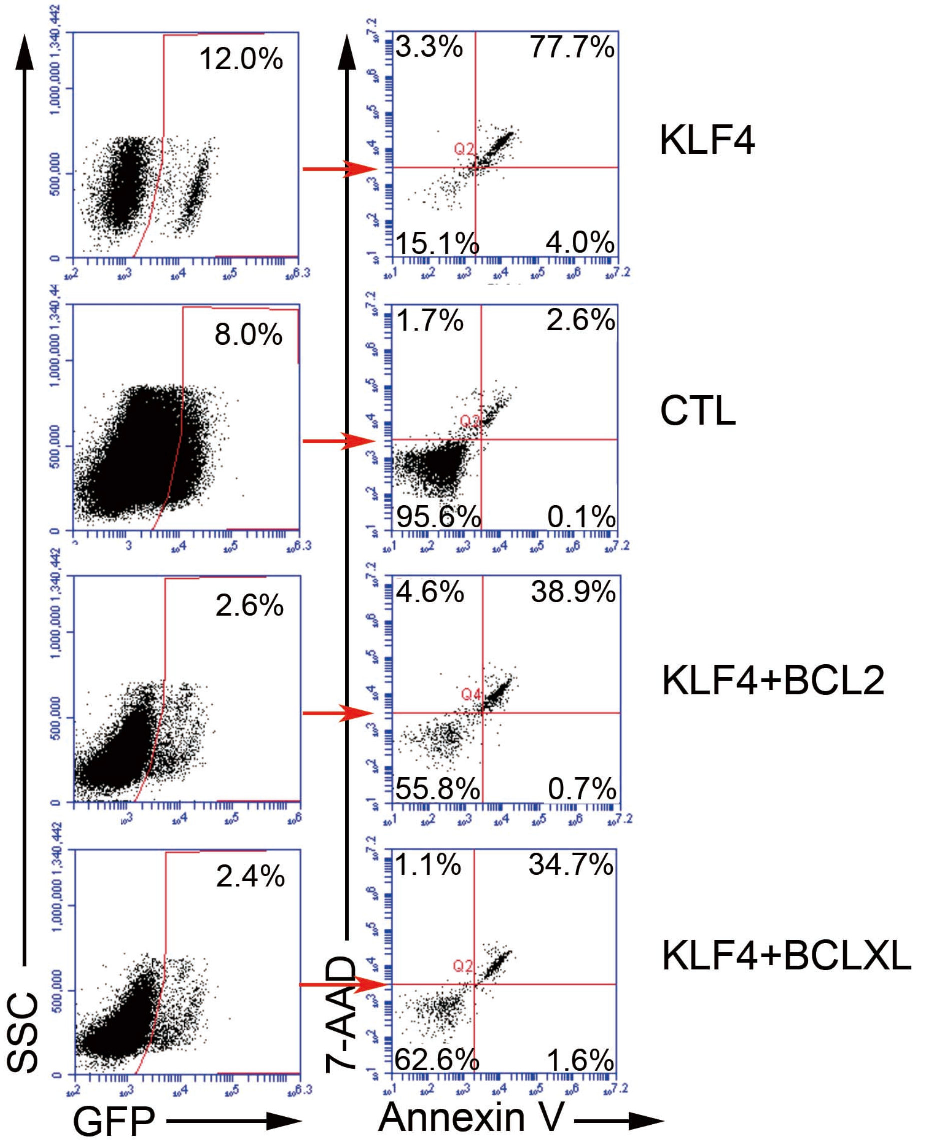

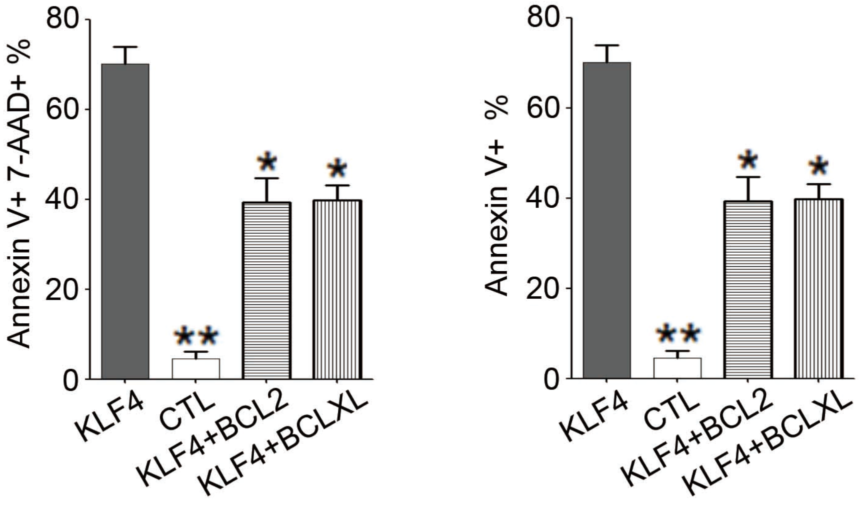

Supplementary Figure 9

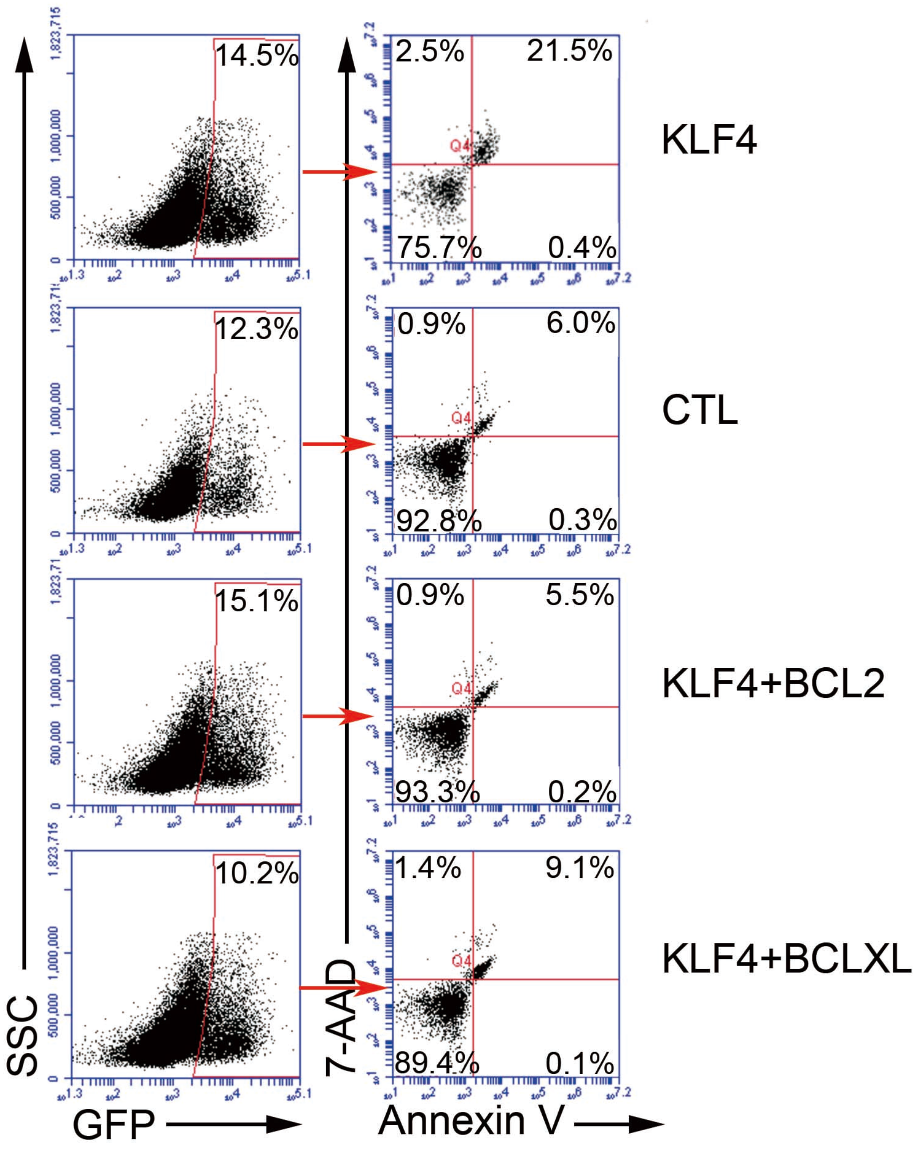

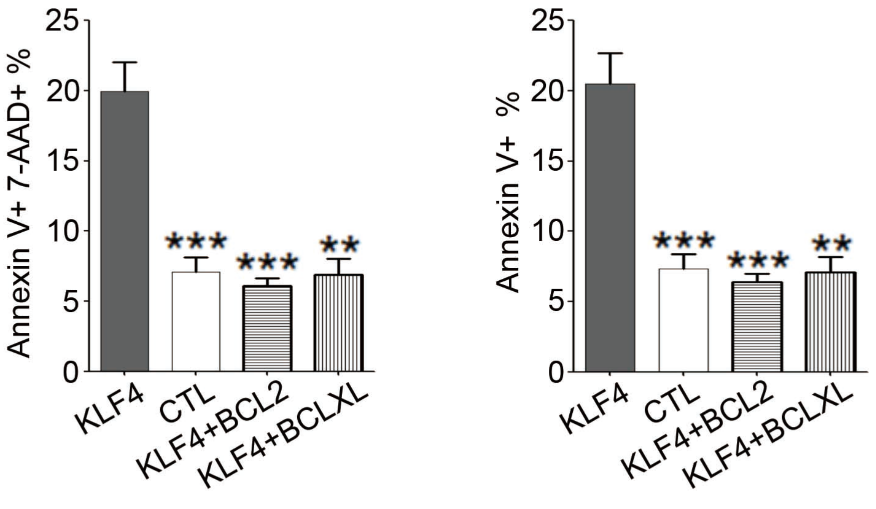

Supplementary Figure 10

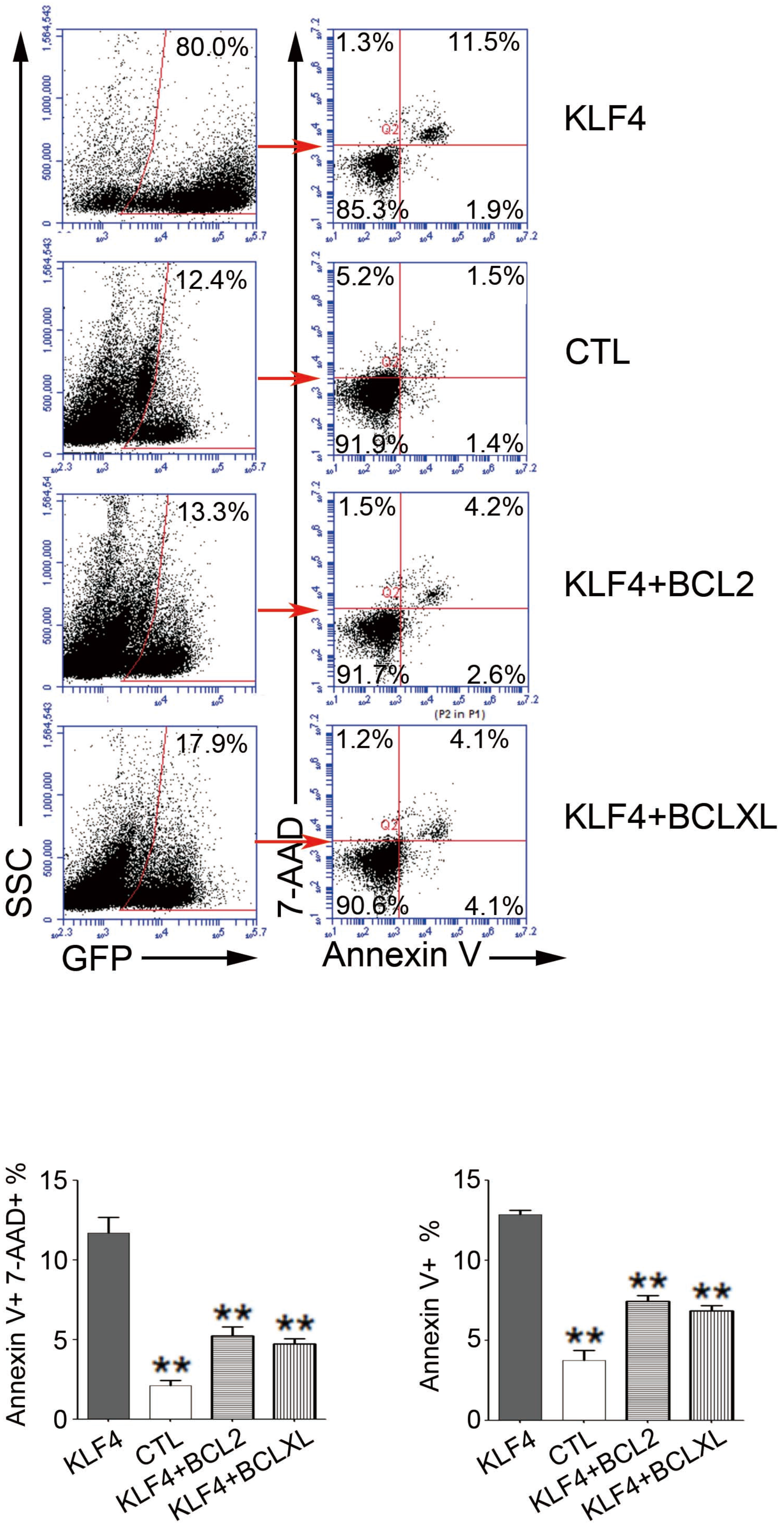

Supplementary Figure 11

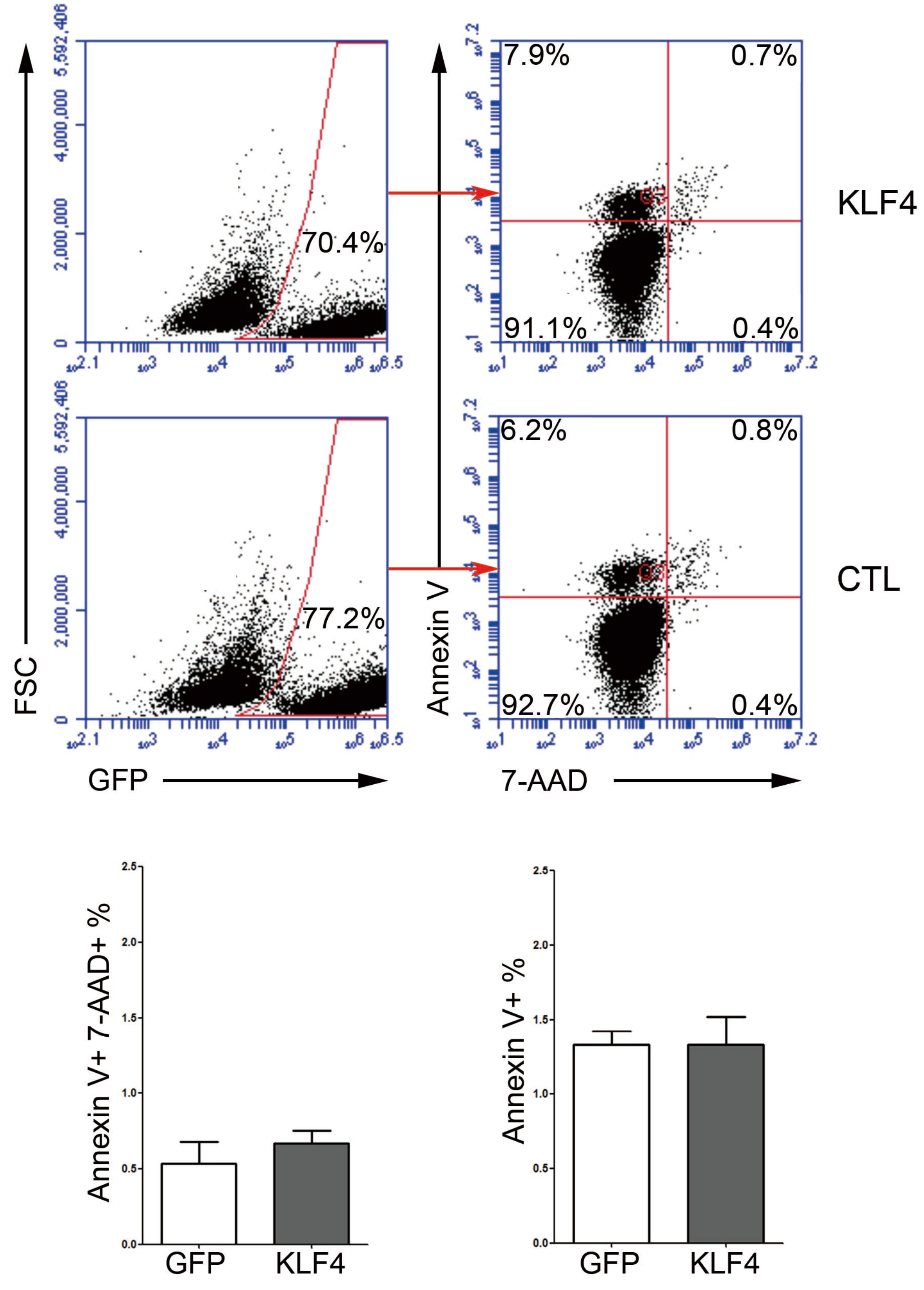

Supplementary Figure 12

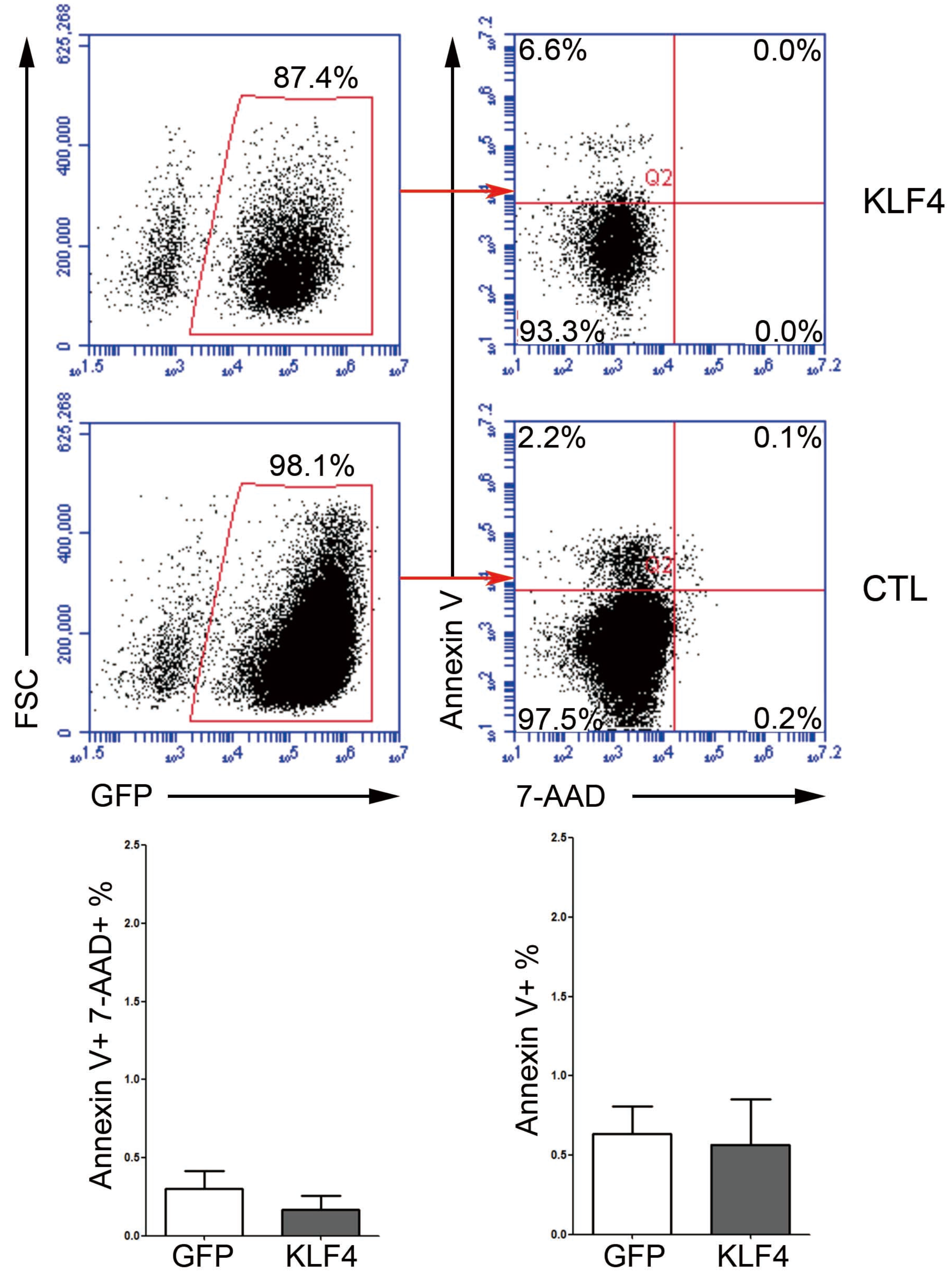

Supplementary Figure 13

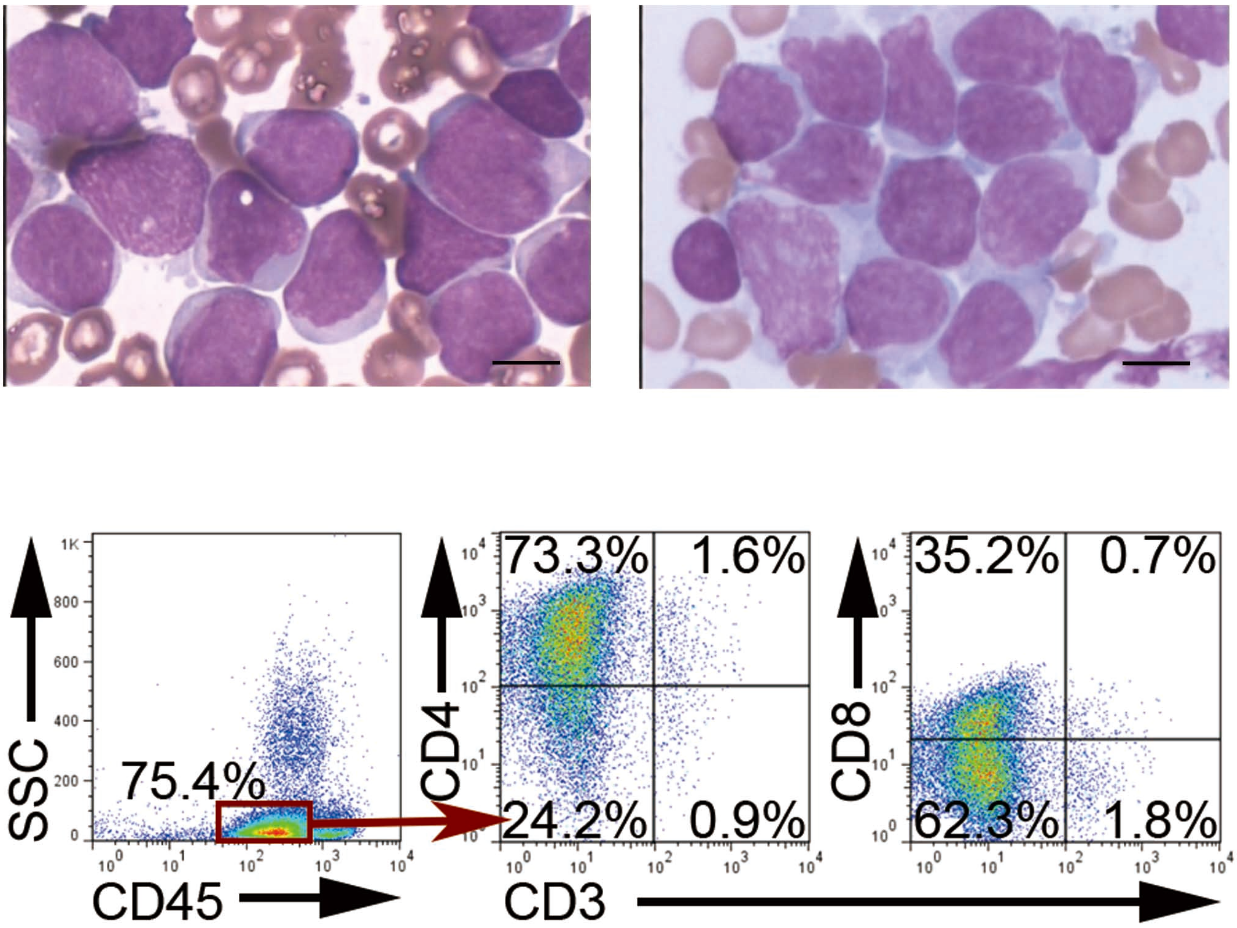

Supplementary Figure 14

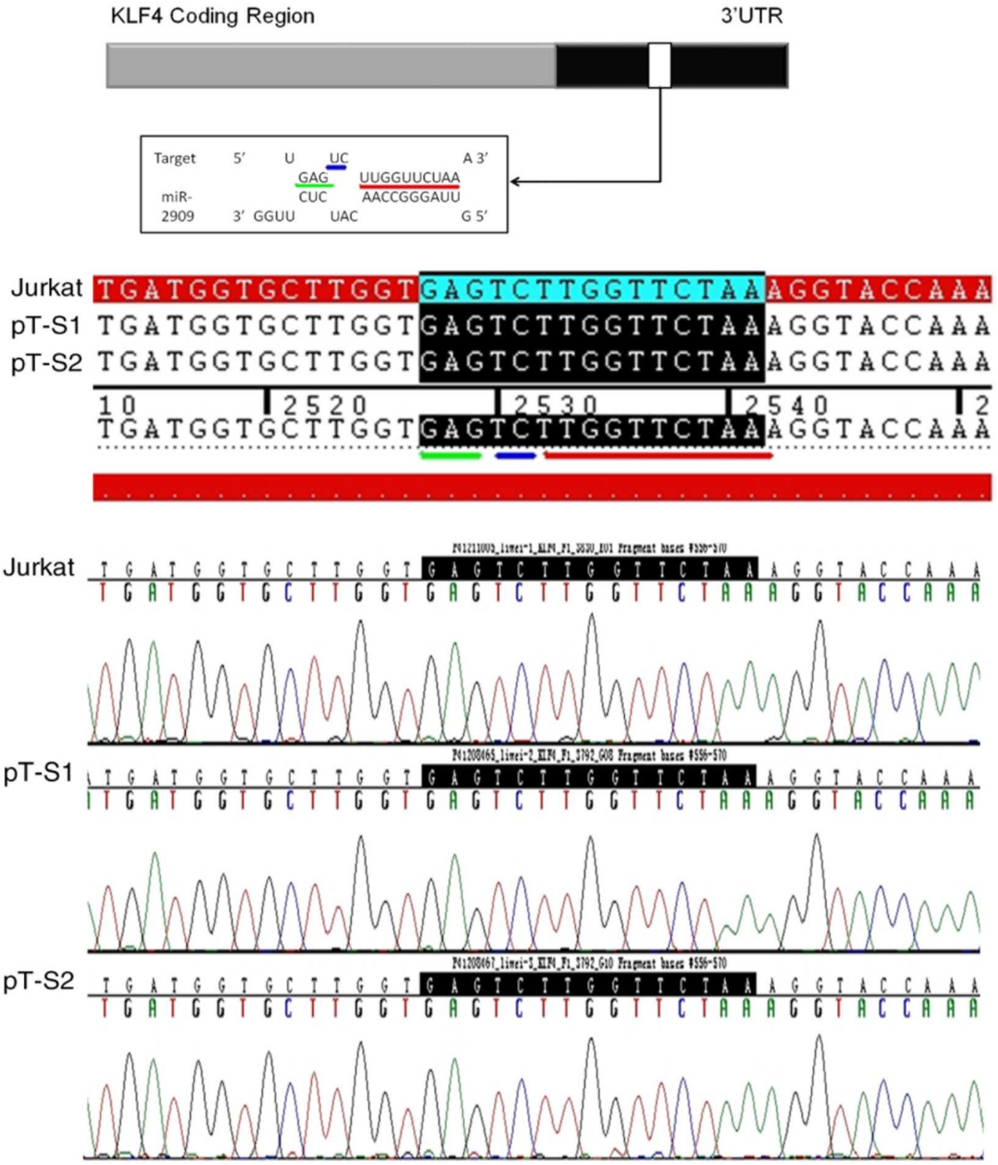

Supplementary Figure 15

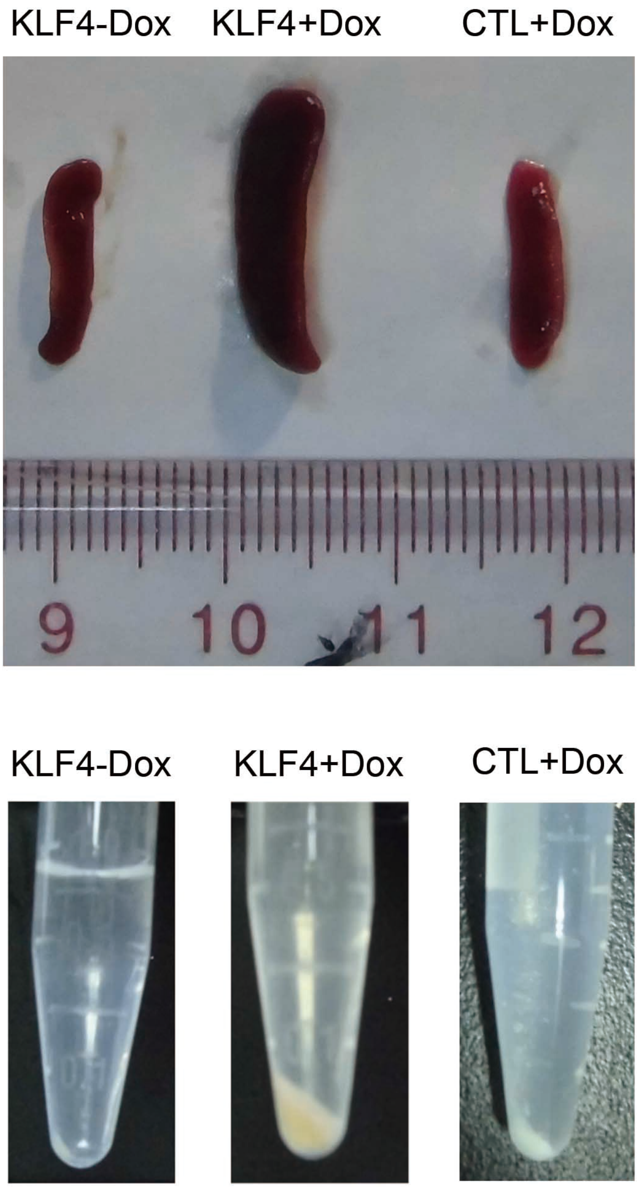

Supplementary Figure 16

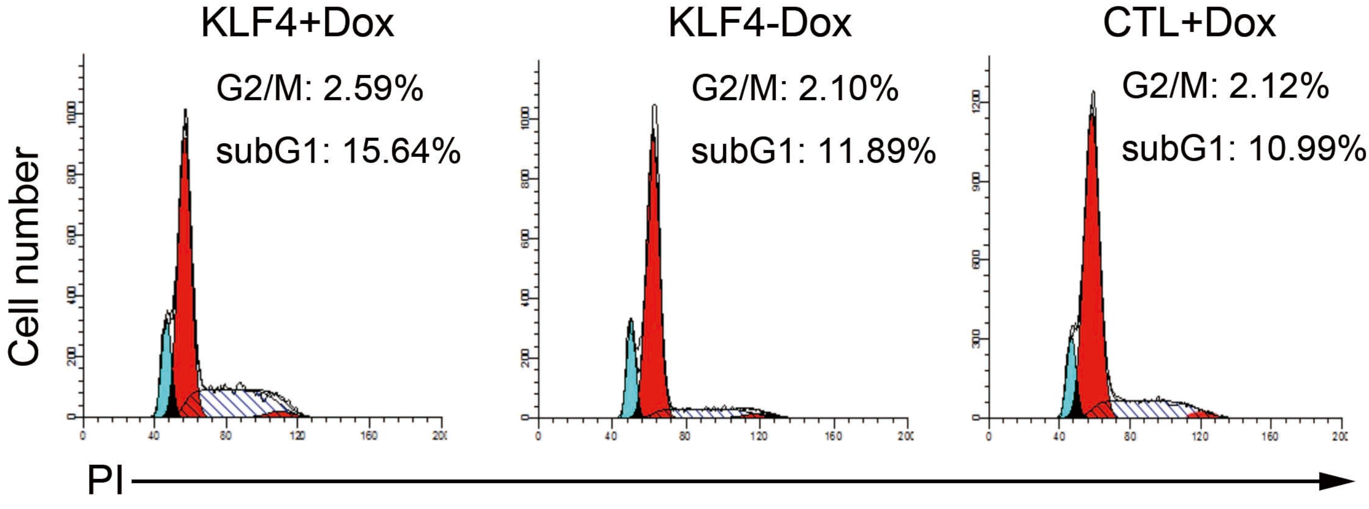

Supplementary Figure 17

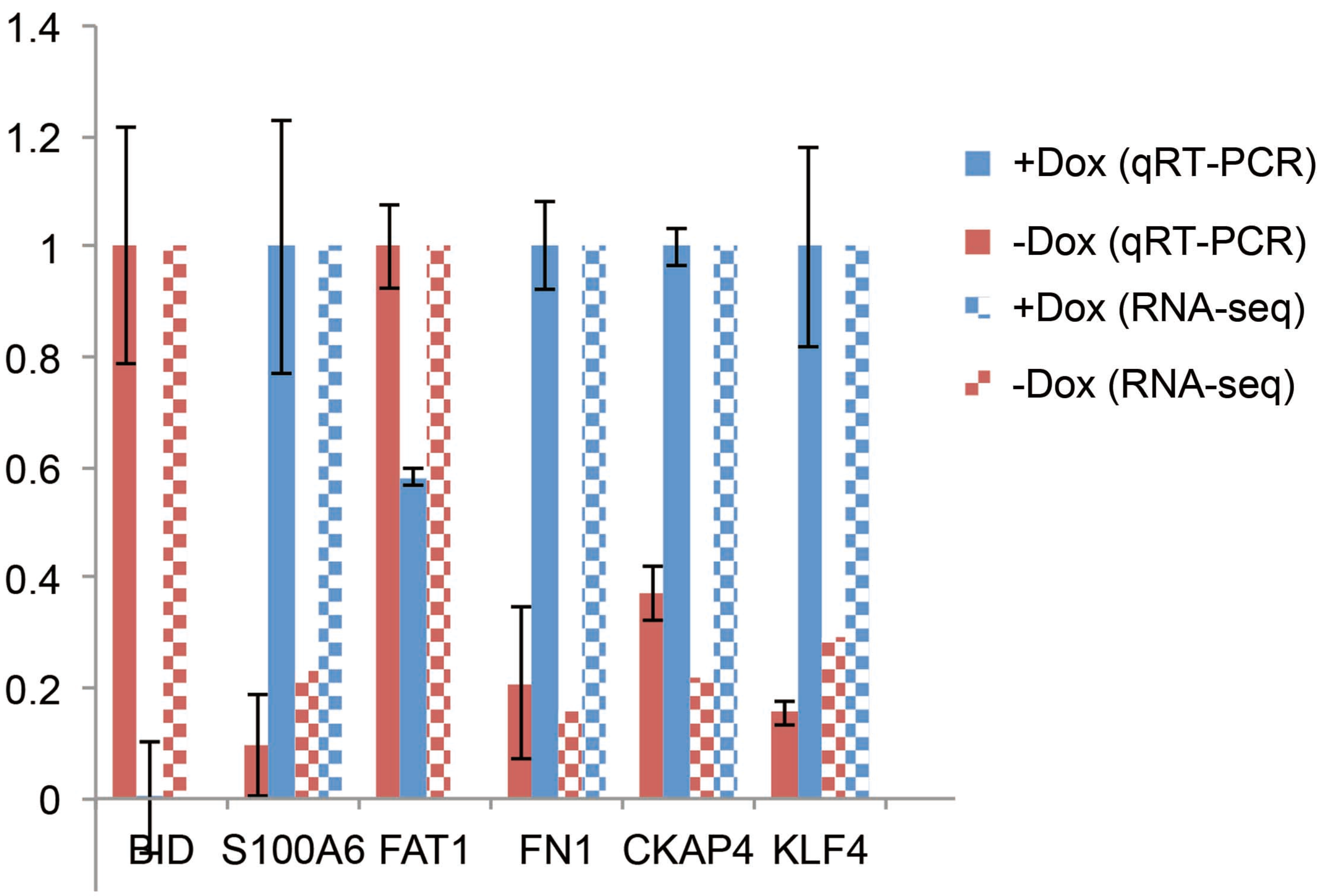

Supplementary Figure 18

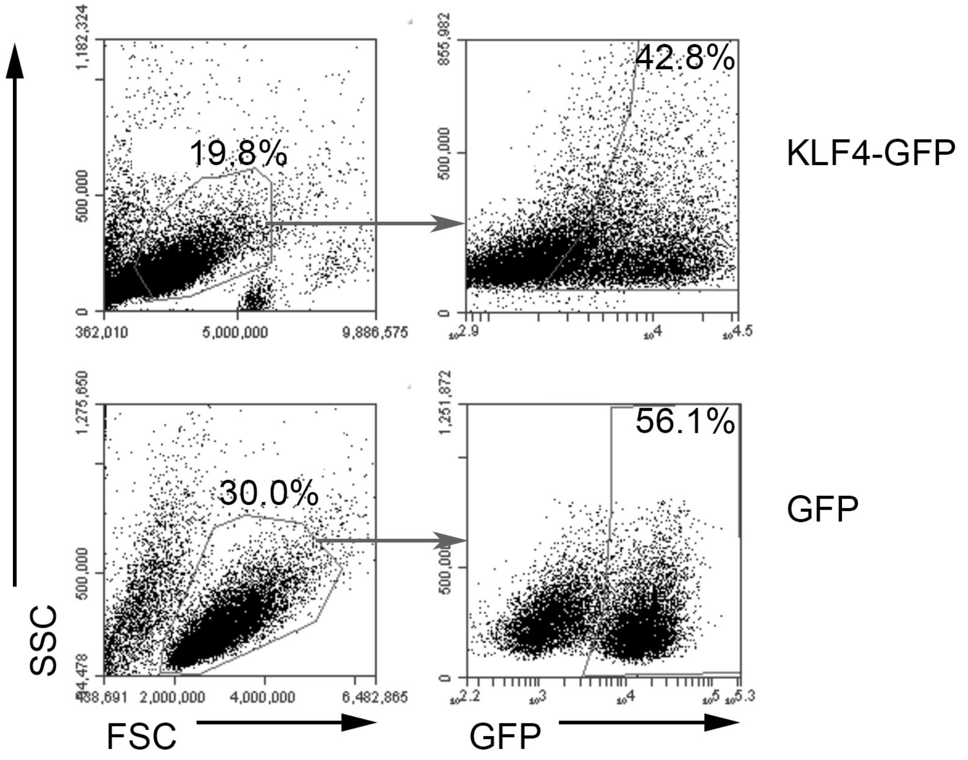

Supplementary Figure 19

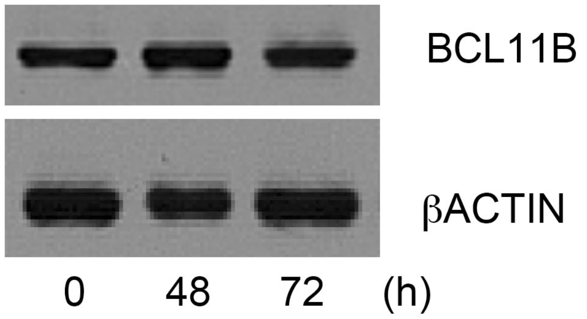

Supplement: Additional file 1: — Supplementary Figures. [file 12943_2014_285_MOESM1_ESM.pdf]
