# Supplementary material for: Genome-wide analyses identify KLF4 as an important negative regulator in T-cell acute lymphoblastic leukemia through directly inhibiting T-cell associated genes
Source: Mol Cancer. 2015 Feb 3;14:26. doi: 10.1186/s12943-014-0285-x (PMC4350611; doi:10.1186/s12943-014-0285-x)
Supplement: Additional file 2: — Supplementary Figure Legends. [file 12943_2014_285_MOESM2_ESM.docx]

**Supplementary Figure 1. Schematic diagram of vectors.** TRE-KLF4: the Dox-inducible KLF4 overexpression lentivirus vector. RTTA-GFP: the reverse tet-controlled transcriptional activator and GFP overexpression lentivirus vector. KLF4-GFP: the KLF4 overexpression lentiviral vector. GFP: the GFP-overexpression lentiviral vector. KLF4-BCL2: the KLF4 and BCL2 overexpression lentiviral vector. KLF4-BCLXL: the KLF4 and BCLXL overexpression lentiviral vector. KLF4-GATA3: the KLF4 and GATA3 overexpression lentiviral vector. KLF4-ICN1: the KLF4 and ICN1 overexpression lentiviral vector. The long terminal repeat (LTR); poly purine tract (ppt), elongating factor 1 α (EF1α); Woodchuck posttranslational regulatory element (W); tet regulatory element (TRE); reverse tet-controlled transcriptional activator (rtTA) are shown.

**Supplementary Figure 2. Establishment of TRE-KLF4 and TRE-empty Jurkat cell lines.** To establish TRE-KLF4 cell line, Jurkat cells were transfected with the TRE-KLF4 and RTTA-GFP lentiviruses. The TRE-KLF4 cell line, in which TRE-KLF4 and RTTA-GFP were genetically integrated, was established from purified GFP+ cells. Similarly, Jurkat cells were transfected with the TRE-empty and RTTA-GFP lentiviruses. The TRE-empty cell line, in which TRE-enpty and RTTA-GFP were genetically integrated, was established from purified GFP+ cells.

**Supplementary Figure 3. Effects of Dox on Jurkat cells.** Quantification of KLF4 and its downstream genes mRNA levels in Jurkat cells with and without Dox treatment. The results were normalized to the GAPDH mRNA levels and are represented as the mean +/- SEM (n=3).

**Supplementary Figure 4. Detection of Caspase-3 degradation.** Caspase-3 was activated after KLF4 overexpression in Jurkat cells. Cytoplastic lysates of KLF4-expressing and GFP-expressing Jurkat cells were analyzed by Western blotting. The non-specific band migrating between the p32 and p17 bands was a loading control.

**Supplementary Figure 5. Depolarization of mitochondrial membrane potential (△ψ_m_) assay with JC-1 in Jurkat cells upon KLF4 overexpression.** Representative flow cytometric plots illustrates that KLF4 overexpression induced △ψm depolarization in Jurkat cells characterized by transformation of JC-1 dye(FL2) aggregates to JC-1 monomers(FL1). Three independent experiments were repeated.

**Supplementary Figure 6. Protein levels of KLF4, BCL2, and BCLXL in Jurkat cells.** To measure protein levels of KLF4, BCL2, and BCLXL, Jurkat cells that were transfected with KLF4, GFP only (CTL), KLF4-BCL2 (KLF4+BCL2), and KLF4-BCLXL (KLF4+BCLXL) lentiviruses were subjected for western blot analysis.

**Supplementary Figure 7. Absence of KLF4 in MOLT4, CCRF-CEM, and CUTLL1 cell lines.** Quantification of KLF4 mRNA levels in three T-ALL cell lines, including MOLT4, CCRF-CEM, and CUTLL1. The results were normalized to the GAPDH mRNA levels and are represented as the mean +/- SEM (n=3).

**Supplementary Figure 8. KLF4-induced apoptosis can be rescued by BCL2 or BCLXL in MOLT4 cell line.**  Top, MOLT4 cells were transduced with either KLF4-GFP (KLF4), GFP (CTL), KLF4-BCL2 (KLF4+BCL2), or KLF4-BCLXL (KLF4+BCLXL) lentiviruses. 48 hours later, GFP-positive cells were subjected to apoptosis assays as measured by Annexin-V binding and 7-AAD staining. Bottom, summary of percentages of apoptotic cells (Annexin-V+7-AAD+ or Annexin-V+) from three independent apoptosis assays. Data are represented as the mean +/- SEM. For Annexin-V+7-AAD+, ***P* ≤ 0.01 versus bar 1 (for bar 2), * *P* ≤ 0.05 versus bar 1 (for bars 3 and 4); Annexin-V+, ***P* ≤ 0.01 versus bar 1 (for bar 2), * *P* ≤ 0.05 versus bar 1 (for bars 3 and 4).

**Supplementary Figure 9. KLF4-induced apoptosis can be rescued by BCL2 or BCLXL in CCRF-CEM cell line.**  Top, CCRF-CEM cells were transduced with either KLF4-GFP (KLF4), GFP (CTL), KLF4-BCL2 (KLF4+BCL2), or KLF4-BCLXL (KLF4+BCLXL) lentiviruses. 48 hours later, GFP-positive cells were subjected to apoptosis assays as measured by Annexin-V binding and 7-AAD staining. Bottom, summary of percentages of apoptotic cells (Annexin-V+7-AAD+ or Annexin-V+) from three independent apoptosis assays. Data are represented as the mean +/- SEM. For Annexin-V+7-AAD+, ****P* ≤ 0.001 versus bar 1 (for bars 2 and 3), ** *P* ≤ 0.01 versus bar 1 (for bar 4); Annexin-V+, ****P* ≤ 0.001 versus bar 1 (for bars 2 and 3), ** *P* ≤ 0.01 versus bar 1 (for bar 4).

**Supplementary Figure 10. KLF4-induced apoptosis can be rescued by BCL2 or BCLXL in CUTLL1 cell line.**  Top, CUTLL1 cells were transduced with either KLF4-GFP (KLF4), GFP (CTL), KLF4-BCL2 (KLF4+BCL2), or KLF4-BCLXL (KLF4+BCLXL) lentiviruses. 48 hours later, GFP-positive cells were subjected to apoptosis assays as measured by Annexin-V binding and 7-AAD staining. Bottom, summary of percentages of apoptotic cells (Annexin-V+7-AAD+ or Annexin-V+) from three independent apoptosis assays. Data are represented as the mean +/- SEM. For Annexin-V+7-AAD+, ***P* ≤ 0.01 versus bar 1 (for bars 2-4); For Annexin-V+, ***P* ≤ 0.01 versus bar 1 (for bars 2-4).

**Supplementary Figure 11. KLF4 overexpression did not induce apoptosis in RL cell line.**  Top, RL cells were transduced with either KLF4-GFP (KLF4) or GFP (CTL) lentiviruses. 48 hours later, GFP-positive cells were subjected to apoptosis assays as measured by Annexin-V binding and 7-AAD staining. Bottom, summary of percentages of apoptotic cells (Annexin-V+7-AAD+ or Annexin-V+) from three independent apoptosis assays. Data are represented as the mean +/- SEM.

**Supplementary Figure 12. KLF4 overexpression did not induce apoptosis in K562 cell line.**  Top, K562 cells were transduced with either KLF4-GFP (KLF4) or GFP (CTL) lentiviruses. 48 hours later, GFP-positive cells were subjected to apoptosis assays as measured by Annexin-V binding and 7-AAD staining. Bottom, summary of percentages of apoptotic cells (Annexin-V+7-AAD+ or Annexin-V+) from three independent apoptosis assays. Data are represented as the mean +/- SEM.

**Supplementary Figure 13. Characterization of primary T-ALLs.** Top, morphology of BM cells from a T-ALL patient was studied under microscopy (Wright-Giemsa). Scale bar, 50μm. Neoplastic cells were found in T-ALL group. Bottom, a representative FACS analysis of mononuclear BM cells from a T-ALL patient.

**Supplementary Figure 14. Sequencing analysis of miR-2909 binding site mutation in Jurkat cells and primary T-ALL cells.** The sequence alignment of the miR-2909 binding site within KLF4 3′UTR region in Jurkat cells and primary T-ALL cells. pT-S1: primary T-ALL Sample1, pT-S2: primary T-ALL Sample2.

**Supplementary Figure 15. Comparison of the size and cellularity of spleens.** Top, a representative picture of spleens from three groups of mice: Dox-untreated mice injected with TRE-KLF4 Jurkat cells (KLF4-Dox), Dox-treated mice injected with TRE-KLF4 Jurkat cells (KLF4+Dox), and Dox-treated mice injected with TRE-empty Jurkat cells (CTL+Dox). Bottom, a representative picture of splenocytes from the three groups of mice as described above after lysis of red blood cells and centrifugation.

**Supplementary Figure 16. Cell cycle was not altered in KLF4 overexpressing Jurkat cells.** Cell cycle profiles of Jurkat cells that were isolated from three groups of mice described in supplementary figure 8 were evaluated by PI staining and flow cytometry analysis. Results are representative of two independent analyses. Percentages of cells in G2/M and sub-G1 are indicated in each panel.

**Supplementary Figure 17. Validation of RNA-seq analysis.** Relative expression levels of six selected genes in Dox-treated and Dox-untreated TRE-KLF4 Jurkat cells were measured by qRT-PCR. The results were normalized to βACTIN mRNA levels and represent the means +/- s.e.m. (n=3). The qRT-PCR results were compared to the expression levels of these six genes in Dox-treated and Dox-untreated TRE-KLF4 Jurkat cells indicated by RNA-seq analysis.

**Supplementary Figure 18. Transduction of KLF4-GFP and GFP-only lentiviruses in Jurkat cells.** Jurkat cells were transduced with KLF4-GFP and GFP-only lentiviruses respectively. After transduced with KLF4-GFP lentivirus, Jurkat cells continuously expressed KLF4 and GFP. Similarly, Jurkat cells constitutively expressed GFP after transduction of GFP-only lentivirus.

**Supplementary Figure 19. Dox itself did not affect BCL11B expression.** Western blot detection of native BCL11B protein in wild-type Jurkat cells after Dox treatment at indicated time points.
