# Supplementary material for: Genome-wide analyses identify KLF4 as an important negative regulator in T-cell acute lymphoblastic leukemia through directly inhibiting T-cell associated genes
Source: Mol Cancer. 2015 Feb 3;14:26. doi: 10.1186/s12943-014-0285-x (PMC4350611; doi:10.1186/s12943-014-0285-x)
Supplement: Additional file 4: Table S1. — Primers used in this study. [file 12943_2014_285_MOESM4_ESM.doc]

**Supplemental Table 1**

**Primers used in this study.**

| Genes | Usage | Forward Primers | Reverse Primers |
| --- | --- | --- | --- |
| KLF4 | Vector construction | GAATTCACCATGAGGCAGCCACCTGGCGAG | ACTAGTGAAAAATGCCTCTTCATGTGTAAGG |
| BCL2 | qRT-PCR | GGTGGGGTCATGTGTGTGG | CGGTTCAGGTACTCAGTCATCC |
| NOTCH1 | qRT-PCR | CGCTGACGGAGTACAAGTG | GTAGGAGCCGACCTCGTTG |
| BCLXL | qRT-PCR | TTGGATCCAGGAGAACGGCGG | ATTTCCGACTGAAGAGTGAGC |
| TRAIL | qRT-PCR | ACTTTACCAACGAGCTGAAGCAG | CTTTCTAACGAGCTGACGGAGTTG |
| GATA3 | qRT-PCR | GCCCCTCATTAAGCCCAAG | TTGTGGTGGTCTGACAGTTCG |
| GAPDH | qRT-PCR | GGAGCGAGATCCCTCCAAAAT | GGCTGTTGTCATACTTCTCATGG |
| BCL11B | qRT-PCR | GGTGCCTGCTATGACAAGG | GGCTCGGACACTTTCCTGAG |
| TCF7 | qRT-PCR | TGCACATGCAGCTATACCCAG | TGGTGGATTCTTGGTGCTTTTC |
| P21 | qRT-PCR | TGTCCGTCAGAACCCATGC | AAAGTCGAAGTTCCATCGCTC |
| TP53 | qRT-PCR | CCCCTCCTGGCCCCTGTCATCTTC | GCAGCGCCTCACAACCTCCGTCAT |
| KLF4 | qRT-PCR | CAGCTTCACCTATCCGATCCG | GACTCCCTGCCATAGAGGAGG |
| KLF4 | Nested-PCR 1^st^ Round | TAGTGTTGGGTATTGTTTTTTT | TTACTATAACAACTAAATCAACAAAC |
| KLF4 | Nested PCR 2^nd^ Round | AGATGGAGGGTTGGATGAGTTA | CAACCAAACAACTAACGAACTA |
| NOTCH1 | ChIP | AACGAGAAGTAGTCCCAGGC | GCACTAGTGAGGCTCAGAGT |
| CXCR4 | ChIP | AACACGAGGATGGCAAGAGA | TGCGGTCTTAAAACGAAGGC |
| BCL2 | ChIP | Tttccagcccttgttttcatg | ttggtggtcgctggggtc |
| βACTIN | ChIP | GACTTCTAAGTGGCCGCAAG | TTGCCGACTTCAGAGCAAC |
| KLF4 S1 | Sequencing | TGACCAGGCACTACCGTAAACACAC | AAAACTTAATTCTCACCTTGAGTATG |
| KLF4 S2 | Sequencing | GGTGAGAAACCTTACCACTGTGACTG | TCACCTTGAGTATGCAAAATACAAAC |
