# Supplementary material for: Genome-wide analyses identify KLF4 as an important negative regulator in T-cell acute lymphoblastic leukemia through directly inhibiting T-cell associated genes
Source: Mol Cancer. 2015 Feb 3;14:26. doi: 10.1186/s12943-014-0285-x (PMC4350611; doi:10.1186/s12943-014-0285-x)
Supplement: Additional file 5: — Supplementary Methods. [file 12943_2014_285_MOESM5_ESM.docx]

**Supplementary Materials and Methods**

**Cell culture**

All T-ALL cell lines were obtained from the American Type Culture Collection (ATCC, Manassas, Virginia, USA) and maintained in RPMI-1640 media (Gibco, New York, USA) with 10% fetal bovine serum (FBS, Hyclone, Utah, USA). The 293T cells used for lentivirus packaging were kindly provided by Professor Duanqing Pei and maintained in DMEM media (Hyclone, Utah, USA) with 10% FBS. OP9-DL1 cells were obtained from Dr. J. C. Zuniga Pflucker (University of Toronto, Toronto, Canada). All cells were incubated at 37°C in 5% CO_2_.

All primary samples were obtained with informed consent for research purposes, and the procedures were approved by the Research Ethics Board of GIBH. For human adult T cells, peripheral blood mononuclear cells (PBMCs) were isolated from the buffy coats of healthy donors. T cells were purified using the Pan T-Cell Isolation kit II (Miltenyi, Bergisch Gladbach, Germany), stimulated with anti-CD3 and CD28 antibodies (BD Pharmingen, San Jose, California, USA ), and cultured in RPMI-1640 medium supplemented with 1 mM sodium pyruvate, 1 mM non-essential amino acids (Invitrogen), 10% FBS (Hyclone), and 20 U/ml recombinant human IL-2 (Peprotech, Rochy Hill, USA). T-ALL clinical samples were obtained with informed consent from donors, and related studies were approved by the Institutional Review Boards at Jinan University Medical School. In all four T-ALL patients, more than 80% of PBMC were T-ALL cells. Cord blood samples were collected at the South China Medical University (SCMU) Department of Gynecology and Obstetrics with informed consent for research purposes only, and this process was monitored by the Institutional Review Boards of SCMU. Adult human bone marrow (BM) samples were collected with informed consent from donors, and the studies involved were approved by the Institutional Review Board of SCMU.

**Lentivirus production and transduction**

A second-generation VSV-G pseudo-typed lentivirus was generated by transiently co-transfecting 293T cells with a combination of three plasmids. Briefly, one 15-cm tissue culture dish containing 293T cells at 70% confluence was transfected with Lipofectamine 2000 (Invitrogen, New York, USA) and 8 µg lentiviral vector, 12 µg pSPAX2, and 4 µg pMD2.G. Supernatants were collected every 24 hours from 24 to 72 hours post transfection and then concentrated by ultracentrifugation, titered, and analyzed after 96 hours using a FACS analyzer (FACS C6, Becton-Dickinson Immunocytometry Systems, San Jose, California, USA). Lentiviral titers ranged from 1×107 to 1×108 TU/mL. For the Dox-inducible system, the lentivirus packaging procedure was performed according to the manufacturer’s protocol. Transduction was performed via ‘spin-infection,’ whereby cells and lentiviruses (cell lines: MOI=1, primary cells: MOI=10) plus 10 μg/mL polybrene were mixed and spun at 900 x g for 2 hours. The transduced cells were then cultured for two days prior to analysis by flow cytometry. For the Dox-regulated Jurkat cell line TRE-KLF4, wild-type Jurkat cells were simultaneously transduced with two lentiviruses encoding rtTA and TRE-KLF4. After transduction, cells were cultured for 7 days and then FACS-purified for GFP+ cells (FACS Aria II, Becton-Dickinson Immunocytometry Systems, San Jose, California, USA).

**Western blot**

Cells were lysed with RIPA buffer (Pierce, Rockford, Illinois, USA), and proteins were quantified using the BCA Protein Assay kit (Pierce, Rockford, Illinois, USA). Samples were loaded onto an 8 to 12% SDS-PAGE gel, blotted to a PVDF membrane, and sequentially probed with primary antibodies. A species-matched HRP-conjugated secondary antibody was then added to detect proteins by autoradiography using an enhanced chemiluminescence kit (ECL Plus, General Electric Healthcare, Little Charfont, UK).

**Growth assay**

TRE-KLF4 Jurkat cells were seeded at a starting density of 1×10^6^ cells/mL in culture medium with or without Dox treatment (2 μg/mL). Cell numbers were determined by live cell counting in triplicate with a hemocytometer every day for up to 6 days.

**Apoptosis assays**

Annexin-V binding assays were conducted at 24, 48, or 72 hours after lentivirus transduction using the Apoptosis Detection Kit (eBioscience, San Diego, California, USA) according to the manufacturer’s instructions. Briefly, cells were washed twice with PBS, resuspended in 100 μL of binding buffer containing APC-conjugated Annexin V, and incubated in the dark for 15 minutes. Cells were then washed and suspended in 200 μL of binding buffer containing 7-AAD. The percentage of apoptotic cells was quantified by FACS analysis.

**Mitochondrial membrane potential assays**

For analysis of the mitochondrial membrane potential, the collected cells were stained with the JC-1 fluorescent probe (Beyotime, C2006, China) and analyzed using a BD Accuri C6 flow cytometer and BD Accuri C6 software.

**DNA sequencing and data analysis**

RNA abstracted from Jurkat cells and 2 primary T-ALL patient sample cells was reverse transcribed to cDNA, and then 3’untranslated region (3’UTR) of KLF4 was amplified by utilizing NEB Q5 polymerase. The PCR resultant products were purified by QIAGEN PCR furification kit and then sent to be sequenced utilizing KLF4 3’UTR sequencing primers (supplementary Table1) based on ABI3730 DNA sequencer platform. The result of sequence data was analysed and blasted by using Sequencher 4.8 Demo software

(<http://softadvice.informer.com/Sequencher_4.8_Demo.html>)

**Image acquisition**

Images were acquired on a Leika DMI6000B fluorescence microscope using 20×objective lens. Images of lentivirus-infected Jurkat cells in RPMI-1640 culture media were acquired at room temperature by DFC digital camera using Leika FW4000 version 1.2.1 software.

**RNA isolation, reverse transcription, and real-time RT-PCR**

Total RNA was isolated from cells using TRIzol reagent (Invitrogen, New York, USA) and cDNA was obtained with the SuperScript reverse transcriptase kit (TransGene, Beijing, China) using oligo-dT primers. All primers were synthesized by Invitrogen (Guangzhou, China). Gene expression was measured with the CFX96 Real-time PCR System (Bio-Rad, Hercules, CA, USA) using the SYBR Select Master Mix (Invitrogen, New York, USA) according to the manufacturer’s protocol. All of the primers used in this study are listed in the supplementary table 1.
